# Vitamin D supplementation among Bangladeshi children under-five years of age hospitalised for severe pneumonia: A randomised placebo controlled trial

**DOI:** 10.1371/journal.pone.0246460

**Published:** 2021-02-19

**Authors:** Fahmida Chowdhury, Abu Sadat Mohammad Sayeem Bin Shahid, Mosharrat Tabassum, Irin Parvin, Probir Kumar Ghosh, Mohammad Iqbal Hossain, Nur Haque Alam, A. S. G. Faruque, Sayeeda Huq, Lubaba Shahrin, Nusrat Homaira, Zakiul Hassan, Zubair Akhtar, S. Mah-E-Muneer, George J. Fuchs, Tahmeed Ahmed, Mohammod Jobayer Chisti

**Affiliations:** 1 International Centre for Diarrhoeal Disease Research, Bangladesh (icddr,b), Dhaka, Bangladesh; 2 Faculty of Medicine, Discipline of Paediatrics, School of Women’s and Children’s Health, UNSW Sydney, Sydney, New South Wales, Australia; 3 Department of Pediatrics, College of Medicine and Departments of Epidemiology and of Preventative Medicine and Environmental Health, College of Public Health, University of Kentucky, Lexington, Kentucky, United States of America; University of Ghana, GHANA

## Abstract

**Introduction:**

Vitamin D is important for its immunomodulatory role and there is an independent association between vitamin D deficiency and pneumonia. We assessed the effect of vitamin D supplementation on the outcome in children hospitalized for severe pneumonia.

**Methods:**

This was a randomised, double blinded, placebo-controlled clinical trial in children aged >2–59 months with severe pneumonia attending Dhaka Hospital, icddr,b. Children received age-specific megadose of vitamin D_3_ (20,000IU: <6 months, 50,000 IU: 6–12 months, 100,000 IU:13–59 months) or placebo on first day and 10,000 IU as maintenance dose for next 4 days or until discharge (if discharged earlier) along with standard therapy. This trial is registered at ClinicalTrials.gov, number NCT02185196.

**Findings:**

We enrolled 100 children in placebo group and 97 in vitamin D group. On admission, 50 (52%) and 49 (49%) of children in vitamin D and placebo groups, respectively were vitamin D deficient. Among children with a sufficient serum vitamin D level on admission, a lower trend for duration of resolution of severe pneumonia in hours [72(IQR:44–96)vs. 88(IQR:48–132);p = 0.07] and duration of hospital stay in days [4(IQR:3–5)vs.5(IQR:4–7);P = 0.09] was observed in vitamin D group compared to placebo. No beneficial effect was observed in vitamin D deficient group or irrespective of vitamin D status.

**Conclusion:**

Age-specific mega dose of vitamin D followed by a maintenance dose shown to have no statistical difference between the two intervention groups, however there was a trend of reduction of time to recovery from pneumonia and overall duration of hospital stay in under-five children with a sufficient serum vitamin D level on hospital admission.

## Introduction

Pneumonia is the foremost cause of childhood morbidity requiring hospitalization and accounting for 16% of all deaths among children below five years of age in lower income countries [1]. In Bangladesh, 13% (around 12,000 among 100 thousand annual deaths) of child deaths below five years of age were due to pneumonia in 2018, and it was the third biggest killer of children in 2017 [2,3]. Globally over the past three decades child mortality rates from pneumonia reduced three folds [4]. However, if the sustainable development goal (SDG) of a reduction of child mortality by two-thirds is to be achieved by 2030, the progress in reducing child deaths due to pneumonia is slower [2,5]. Pneumonia related deaths remain high in many hospitals in low income countries [6,7] even with appropriate treatment as recommended by the World Health Organization (WHO) [8]. Most deaths due to pneumonia are preventable. Thus, development of effective new interventions is essential to reduce global burden of childhood mortality due to pneumonia.

Studies have suggested an independent association between subclinical vitamin D deficiency with the incidence and severity of acute respiratory tract infections (ARIs) in children [9–15]. The major circulating vitamin D metabolite, 25-hydroxy vitamin D [25(OH)D], supports induction of antimicrobial peptides that boosts mucosal defenses suggesting protection against respiratory pathogens [16]. It has been reported that 1,25 dihydroxy vitamin D [1,25(OH)_2_D], the active metabolite of vitamin D, is important for promoting and regulating immune responses in vitro, and this phenomena has been observed in a number of studies in humans [17–19]. Vitamin D enhances innate immunity through initiation of monocyte differentiation, inhibition of lymphocyte proliferation, stimulation of phagocytosis and antibody dependent macrophages, and modulation of cytokines and antibody production by T and B lymphocytes [17,19–21]. Moreover, severe vitamin D deficiency often leads to chest wall deformity and resultant atelectasis, fibrosis, and hypotonia that can contribute to higher incidence and adverse outcomes of pneumonia [22]. In addition, mortality due to sepsis in children with vitamin D deficiency has been observed to be high [23]. Despite profuse exposure to sunlight, the prevalence of vitamin D deficiency among Bangladeshi children is high [24].

In recent years, several studies have investigated the therapeutic efficacy of vitamin D supplementation to decrease the frequency and severity of ARI in children [21,25–30]. Nonetheless, it is not yet defined whether or not the addition of oral vitamin D supplementation to standard treatment of pneumonia in under-five children results in improved outcomes. All these studies had significant methodological differences or limitations including eligibility criteria and differences in dose and duration of vitamin D supplementation. Four studies used a single mega dose (100,000 IU) of vitamin D supplementation, two studies used a low dose (1000/2000 IU) of vitamin D for 5 days, and one study supplemented with 50,000 IU of vitamin D for 2 days to investigate potential therapeutic efficacy, but none of the studies observed a beneficial effect. However, all the studies evaluated the efficacy of vitamin D with its conventional doses; none of the studies tested the efficacy of high dose vitamin D followed by a maintenance dose in children hospitalized for pneumonia. We therefore designed the current clinical trial to evaluate the impact of an age-appropriate mega dose of vitamin D supplementation followed by a maintenance dose of vitamin D as an adjunct therapy together with standard antibiotic therapy among hospitalized under-five children with severe pneumonia.

## Materials and methods

### Study design, study site and study population

This was a randomised, double blinded, placebo controlled clinical trial (RCT) conducted at the Dhaka Hospital of the International Centre for Diarrhoeal Disease Research, Bangladesh (icddr,b), Dhaka, Bangladesh. This trial is registered at ClinicalTrials.gov, number NCT02185196. Dhaka hospital has a separate acute respiratory infection (ARI) ward for management of children with severe pneumonia and also an Intensive Care Unit (ICU), equipped with mechanical ventilators, cardiac monitors, and other supportive facilities for the management of critically ill children. Children aged >2–59 months, attending the study hospital with clinically diagnosed severe pneumonia comprised the study population. Children were enrolled in the study by the study physician after fulfillment of eligibility criteria and written informed consent by the caregiver of the study participant.

### Exclusion criteria

We excluded children with known case of hypercalcaemia, allergy to vitamin D, congenital heart disease, renal or hepatic insufficiency, hypernatraemia, tuberculosis, or asthma, and critically ill children requiring ICU care, such as those with septic shock, cardiac arrest, or apnoea. Children who received vitamin D or calcium supplementation within four weeks prior to enrolment into the study or with a baseline ionized calcium level (normal range: 1.15–1.33 mmol/L) above the normal limit on admission were also excluded.

### Study intervention

Eligible children were randomized in equal numbers to one of two masked parallel intervention groups, to receive the vitamin D_3_ (cholecalciferol) supplementation or matched placebo in their diet in addition to standard therapy (appropriate antibiotic and supportive therapy). Infants aged 3 to <6 months received breast milk and/or infant formula, and those ≥6 months received complementary food such as “Milk Suji” as their diet [31]. For exclusively breast fed babies, vitamin D_3_/placebo was administered in expressed breast milk. If the baseline ionized calcium was within normal range, vitamin D_3_ or placebo was administered within two hours of the first dose of parenteral antibiotics on the day of admission as well as with the first morning meal or feeding on days 2 to 5 of hospitalisation.

Vitamin D_3_ was a high-concentration (20,000 IU D_3_ per mL) liquid formulation (Vigantol Oil, Merck KGaA, Germany), and the placebo was miglyol oil 812 (Sasol, Germany), the vehicle used in Vigantol Oil. These active supplements and selected placebo were identical in appearance and taste.

### Vitamin D dosing

Mega dose of 20,000 IU vitamin D_3_ in children <6 months, 50,000 IU in children 6–12 months and 100,000 IU in children 13–59 months of age on first day and thereafter 10,000 IU as maintenance dose daily for all children for subsequent four days or till discharge (if discharged earlier).

### Randomisation and allocation concealment

We developed a computer generated random list by a person not involved in the study. The eligible children were allotted a sequential study number, which was previously assigned to vitamin D_3_ or placebo concealed as “A” and “B” in accordance with the randomisation. Study participants and the research staff were blinded to allocation. Pharmaceuticals company prepared the supplements off-site using individual opaque glass vials labeled with concealed unique identifiers as “A” and “B” for vitamin D_3_ and placebo. Allocation concealment for vitamin D3 and placebo was done by a person in the pharmaceuticals company not related with the study in a sealed envelope and was kept in a secured place by a person not associated with the study; the envelope was opened after the data analysis to identify the treatment (vitamin D3/placebo) received as “A” and “B” group.

### Definition of severe pneumonia

Children aged >2–59 months were diagnosed with severe pneumonia if they had a history of cough and/or respiratory difficulty plus oxygen saturation < 90% or central cyanosis, or grunting, or signs of pneumonia with a general danger sign (inability to breastfeed or drink, lethargy or reduced level of consciousness, convulsions), auscultatory findings of diminished or bronchial breath sounds or signs of pleural effusion or empyema [32].

Children who presented with severe malnutrition (children with nutritional oedema or Z scores below -3 standard deviations (SD) from the median for weight for height or weight for age or height for age) with any sign of pneumonia (any of the WHO defined signs of pneumonia or severe pneumonia or radiological pneumonia) was considered as severe pneumonia [33]. A radiologist not involved in patient care interpreted all chest radiographs. All enrolled patients had an admission chest radiograph.

### Collection of baseline information

Relevant information including medical history, socio-demographic characteristics, feeding history, immunisation status, history of contact for tuberculosis, recent respiratory tract infection of any family members and past history of child’s pneumonia, and history of exposure of the child to sun was recorded. Clinical examination findings were recorded including mental status (normal, irritable, lethargic), pulse and respiratory rate, axillary temperature, anthropometric measurement, chest wall in drawing, oxygen saturation, presence of cyanosis, and chest auscultatory findings. Fever was defined as an axillary temperature 38°C or greater. Respiratory rate was counted for full 60 seconds by exposing the trunk when the child was awake and calm, and presence of lower chest wall in-drawing was noted at the same time. Respiratory rate was counted twice and, if rates differed by more than five breaths per minute, a third reading was made and the average of the two closest respiratory rates (not deviating by five or greater number) was recorded as the actual rate. Oxygen saturation was measured using a pulse oximeter (Nellcor Puritan Bennett Inc. N-560, Made in Korea) with a probe on a finger or toe with the child on room air. Hypoxemia was defined as SpO_2_<90% in room air, which was the indication for immediate oxygen therapy.

Chest X-ray was done after enrollment and at discharge as well as at other times if clinically indicated. Five milliliter (5.0 mL) of venous blood was collected from the children just after enrollment for a complete blood count, C reactive protein, blood glucose, electrolytes, “25(OH) D” level, ionized Ca, inorganic phosphorus, alkaline phosphatase, as well as blood culture for bacterial pathogen before the introduction of intravenous antibiotics; 2 ml venous blood was collected at discharge for a serum “25(OH) D” and ionized Ca level assay. A vitamin D level <50nmol/L was considered as vitamin D deficiency and > = 50 nmol/L as sufficient [34]. We also monitored serum parathormone (PTH) level for every fifth patient on admission and at discharge.

### Monitor ionized calcium level instead of total serum calcium

Serum ionized (free) calcium rather than total calcium was measured because malnourished children can have a low albumin level; an ionized calcium therefore more accurately reflects true calcium status. We considered the upper limit (normal range: 1.15–1.33 mmol/L) [35] of ionized calcium for safety monitoring; children with a baseline ionized calcium above 1.33mmol/L on admission were excluded from the study.

### Treatment of severe pneumonia

After the collection of blood specimen, children were treated according to standard treatment protocol for pneumonia followed in the Dhaka Hospital of icddr,b. Children were treated with parental antibiotics ampicillin and gentamicin as first-line treatment. However, children who did not respond (no improvement with 48 hours or presented deterioration of clinical sign symptoms of pneumonia after 24 hours of initiation of antibiotics) antibiotics were switched to second-line antibiotics ceftriaxone and levofloxacin according to Dhaka hospital protocol.

### Primary outcome measure

Our primary outcome was time to resolution of severe pneumonia in hours.

Time to resolution of severe pneumonia was defined as absence of all signs of pneumonia, such as cyanosis, hypoxaemia or lethargy, convulsion and inability to feed, no fever, or lower chest wall in-drawing, or tachypnoea (RR<40 breaths/minute in children above 1 year and RR<50 breaths/minute in children below 1 year) for at least 24 hours and the child could be fed orally.

### Secondary outcome measure

Secondary outcome measures were duration of hospital stay in days, time to resolution of tachypnoea, lower chest wall in-drawing, and hypoxaemia; a new episode of pneumonia defined as an episode within the six months follow up period after discharge.

### Monitoring during hospital stay

During the hospital stay, data were recorded 8 hourly including presence of cough, pulse and respiratory rates, axillary temperature, presence/absence of lower chest wall in-drawing, chest auscultatory findings, oxygen saturation, and feeding.

If the clinical condition deteriorated (e.g., development of septic shock, apnoea [cessation of respiration for 10–15 seconds] or respiratory failure requiring the support of bubble CPAP or mechanical ventilation) at any time of the study, the child was transferred to ICU for appropriate treatment and the vitamin D_3_/placebo intervention was discontinued. Proportion of such children in both the study groups was compared during data analysis.

The amount of vitamin D_3_ received by the patient was also quantified according to dietary source (infant formula/milk suji) and total volume of milk consumed during the hospital stay.

### Criteria for discharge

Discharged criteria were set as resolution of severe pneumonia with establishment of oral feeding and absence of co-morbidities requiring hospitalised treatment.

### Six months follow-up after discharge

Study physicians followed the enrolled children on a weekly basis over cell phone up to 24 weeks after discharge. They asked the caregiver/mother for any symptoms of new episode of acute respiratory illness and categorized them as ‘no acute respiratory illness’, or ‘no pneumonia (cough and cold)’, or ‘pneumonia’, or ‘severe pneumonia’ and/or other illness and recorded them accordingly. A new episode of pneumonia was diagnosed if an episode of pneumonia occurred after 14 days from the first day of resolution of last episode of pneumonia.

### Sample size calculation and statistical analysis

Based on patients records of children hospitalized at the Matlab Hospital of icddr,b, we calculated duration of severe pneumonia (mean = 6, standard deviation = 2.5) [36]. To detect one day difference (standard deviation of 2.5) for resolution of severe pneumonia between the groups with 5% type 1 error, and 90% power, we estimated a needed total sample size of 216 (108 patients were required in each group). However, due to resource constrain we were not able to complete our target sample size and were able to enroll 197 children with severe pneumonia (100 patients in placebo and 97 in vitamin D group). Here it is important to note that if we would consider 5% error and 80% power our target sample size would be 200 (100 patients in each group).

We entered data using SPSS for Windows version 20.0 (SPSS Inc, Chicago, IL), and analyzed it using STATA version 13. The Chi-square test was used to compare categorical variables and independent t test to compare quantitative variables. Intention to treat (ITT) analyses was done for the outcome variables to assess the impact of vitamin D supplementation and deaths with missing resolution were considered as non-resolved for the ITT analysis. We performed stratified analysis between malnutrition and vitamin D groups to evaluate the effect modifier in the treatment effect. To estimate the treatment effect, we adjusted hazard ratio (HR) for age as a potential confounder, Cox proportional hazard regression model was done. We adjusted for age, as children aged 12 months or older had resolved pneumonia earlier than younger infants (median duration in hours: 64 vs. 72; p = 0.02). Hospital duration for children aged 12 months or older compared with children younger than 12 months was shorter (median duration in days: 4 vs. 5; p = 0.02). Since 68% of the children in the study were infants, all hazard calculated modeling were controlled for age as a continuous variable. A p value <0.05 was considered as significant. Risk ratio (RR) was estimated to compare the recurrence of new episode of pneumonia in both the groups.

### Ethical consideration

The study protocol was reviewed and approved by the institutional review boards (IRB; named as Research Review Committee and Ethical Review Committee) of International Centre for Diarrhoeal Disease Research, Bangladesh (icddr,b). CDC relied on icddr,b’s IRB review. We obtained written, informed consent from caregivers/parents of the participating children before enrollment.

## Results

**Fig 1:**
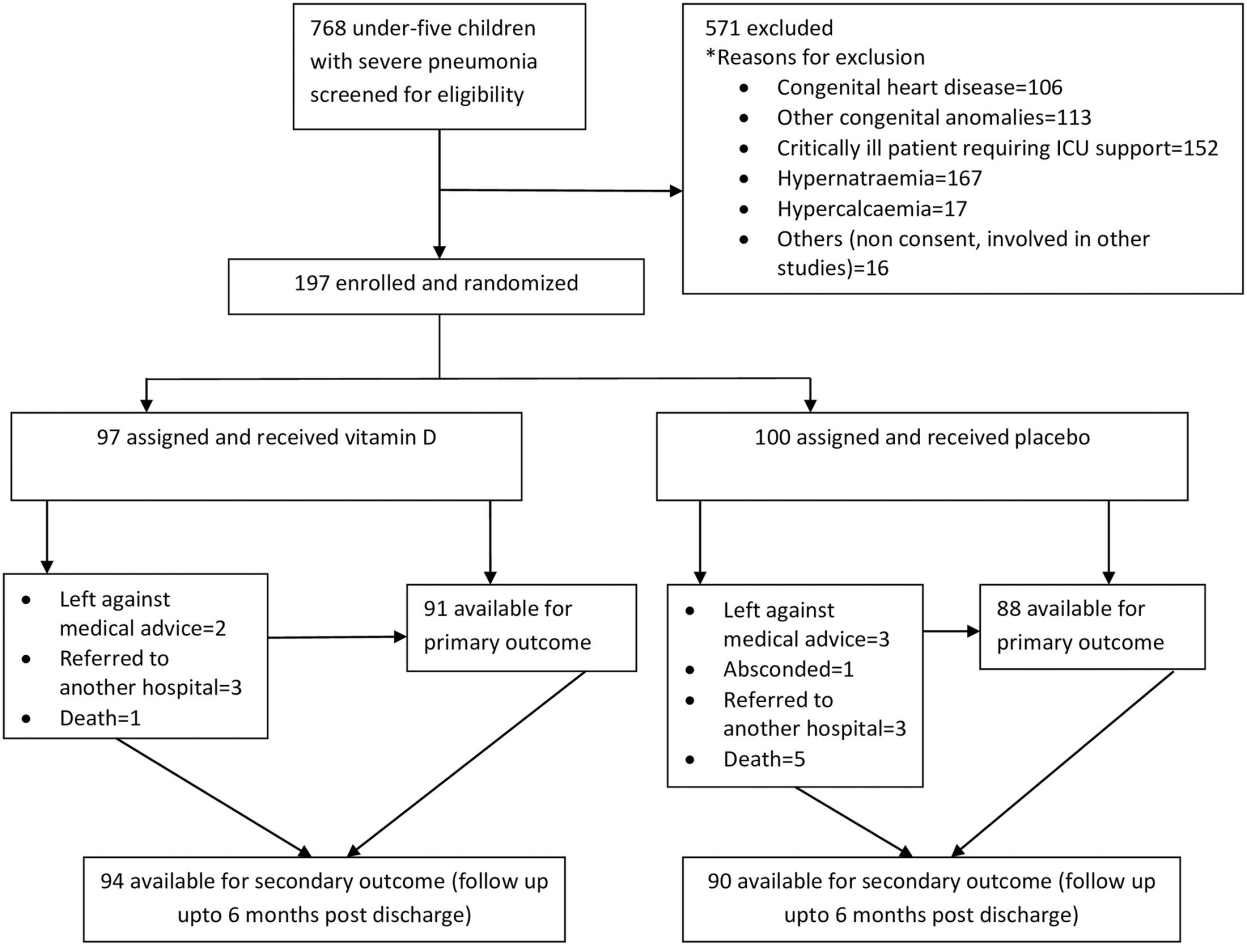
Vitamin D trial profile showing participant enrollment.

The study was conducted from June 2014 to June 2018, during which 197 children (100 in placebo group and 97 in vitamin D group) were enrolled (Fig 1). Of the enrolled children, 134 (68%) were infants aged <12 months and 124 (63%) were male. Approximately half of the children in both groups were severely malnourished (Table 1). By caregiver’s report, 40% and 51% of children in vitamin D group and placebo group, respectively were exclusively breast fed per WHO case definition and 35% children in vitamin D group and 40% children in placebo group had at least one previous episode of pneumonia (Table 1). There were no differences in socio-demographic characteristics between the groups and the baseline characteristics were comparable (Table 1).

**Table 1:** Baseline characteristics by treatment groups among under-five children with severe pneumonia in an urban hospital, Bangladesh.

Characteristics	Vitamin D (N = 97)	Placebo (N = 100)
**Socio-demographic characteristics**		
Age in months, (median, IQR)	8 (5.0, 13.0)	10 (5.5, 14.0)
Male, **n (%)**	65 (67.0)	59 (59.0)
Severe underweight (Z score <-3 SD), **n (%)**	55 (56.7)	58 (58.0)
Severe wasting (Z score <-3 SD), **n (%)**	24 (24.7)	34 (34.0)
Nutritional oedema, **n (%)**	11 (11.3)	8 (8.0)
Breast feeding, **n (%)**	77 (80.2)	71 (71.7)
Exclusively breast fed, **n (%)**	31 (40.3)	36 (50.7)
Maternal age (median, IQR)	23 (20–27)	23 (20–26)
Maternal education, **n (%)**		
*No formal education*	22 (22.7)	18 (18.0)
*1–5 yrs of schooling*	33 (34.0)	48 (48.0)
*>5 yrs of schooling*	42 (43.3)	34 (34.0)
Past history of pneumonia, **n (%)**	34 (35.1)	40 (40.0)
Daily outdoor activity (sun exposure), **n (%)**		
*<0*.*5 hour*	18 (31.0)	12 (19.1)
*>0*.*5 hour*	40 (69.0)	51(81.0)
60% of body covered with clothes while outdoor, **n (%)**	58 (59.8)	63 (63.0)
Single room in family, **n (%)**	63 (65.0)	68 (68.0)
Monthly income (Mean, SD)	11,050.0 (5028.0)	12,710.0 (7879.0)
**Clinical and laboratory characteristics**		
Duration of illness (days) (Median, IQR)	5 (4.0, 7.0)	5 (3.0, 6.5)
Pulse (rate/min) (Mean, SD)	142.2 (11.2)	144.6 (11.8)
Axillary temperature (°C) (Mean, SD)	37.9 (0.8)	38.0 (0.9)
Respiratory rate (breath/min (Mean, SD)	56.9 (8.5)	57.6 (9.0)
Oxygen saturation (SpO_2_) (Mean, SD)	93.5 (5.7)	93.0 (6.9)
Hypoxaemia (SpO_2_< = 90%), **n (%)**	3 (3.1)	3 (3.0)
Axillary temperature >37·8°C, **n (%)**	55 (56.7)	65 (65.0)
Lower chest in-drawing, **n (%)**	61(62.9)	58 (58.0)
Crackles on chest auscultation, **n (%)**	86 (88.7)	88 (88)
Rhonchi on chest auscultation, **n (%)**	6 (6.2)	9 (9)
Grunting, **n (%)**	12 (12.4)	13 (13.0)
Vomiting, **n (%)**	2 (2.1)	3 (3.0)
Less active to lethargic, **n (%)**	7 (7.2)	8 (8.0)
Diarrhoea, **n (%)**	57 (58.8)	48 (48.0)
Dehydration, **n (%)**	3 (5.3)	7 (14.9)
Received antibiotic before admission, **n (%)**	66 (68.0)	61(61.0)
Vitamin D received through diet in IU (Mean, SD)	3596.5 (2383.0)	4117.9 (2833.9)
Serum Vitamin D (nmol/L) (Mean, SD)	53.7 (30.8)	54.1(29.0)
Serum ionized calcium (mmol/L) (Mean, SD)	1.1(0.1)	1.1(0.1)
Serum parathormone (mmol/L) (Mean, SD)	80.1(37.6)	82.8 (34.9)
Serum inorganic phosphorus (mmol/L) (Mean, SD)	1.5 (0.4)	1.5 (0.4)
Serum alkaline phosphatase (mmol/L) (Mean, SD)	242.5 (148.7)	238.7 (125.4)
CRP (mg/dl) (Median, IQR)	1.5 (0.35, 4.1)	1.4 (0.3,4.0)
Blood culture isolates	5 (5.2)	4 (4)
Vitamin D deficient	50 (52)	49 (49)

Median duration of illness at the time of enrollment was 5 days in both the treatment groups (Table 1). Clinical characteristics of pulse rate, temperature, respiratory rate, oxygen saturation, lower chest wall in-drawing, chest auscultatory findings, grunting, vomiting, lethargy, and diarrhoea and dehydration were similar in both groups (Table 1). The amount of dietary vitamin D from feeds during the hospitalization was 3597 IU ± 2383 and 4118 IU ± 2834 in the Vitamin D and placebo groups, respectively. On admission, 50 (52%) children in vitamin D group and 49 (49%) children in placebo group were vitamin D deficient. Laboratory parameters at admission as serum vitamin D level, ionized calcium, parathormone, inorganic phosphorus, alkaline phosphatase and CRP on admission were comparable between the two groups (Table 1).

The vitamin D concentration between the groups at admission were comparable (53.7 nmol/L and 54.1 nmol/L in the vitamin D and placebo groups, respectively, p = 0.93) however, at discharge, the vitamin D concentration was significantly greater in the vitamin D compared to placebo group (93.2 nmol/L vs. 63.1 nmol/L, p = <0.001). Regarding adverse outcome, one child died in vitamin D group and five children died in placebo group during hospitalisation, however, the difference was not statistically significant (Table 2).

**Table 2:** Laboratory characteristics at discharge and outcomes by treatment groups among under-five children with severe pneumonia in an urban hospital, Bangladesh.

Characteristics	Vitamin D (N = 97) N (%)	Placebo (N = 100) N (%)	P-value
**Clinical and laboratory characteristics at discharge**			
Serum Vitamin D (nmol/L) (Mean, SD)	93.22 (42.1)	63.1 (29.1)	<0.001
Serum ionized calcium (mmol/L) (Mean, SD)	1.2 (0.1)	1.1(0.1)	0.12
Serum parathormone (mmol/L) (Mean, SD)	80.0 (37.8)	84.4 (33.4)	0.39
Vitamin D deficient	20 (21)	35 (35)	0.02
**Outcome at discharge**			
Improved and discharged	91(93.8)	88 (88.0)	0.16
**Other outcomes**			
Discharge on risk bond (DORB)	2 (2.1)	3 (3.0)	0.68
Absconded	0 (0.0)	1(1.0)	0.32
**Adverse outcomes**			
Death	1(1.0)	5 (5.0)	0.11
Referred to other hospital	3 (3.1)	3 (3.0)	0.97
**After completion of 6m follow-up**	**N = 94**	**N = 90**	
Death	3 (3.2)	1(1.1)	0.33

Of the children in vitamin D group, 97 (100%) had findings consistent with WHO defined radiological pneumonia [6 (6.2%) had primary end point consolidation, and 91 (93.8%) had other infiltrates]; the placebo group 98 (98%) had findings consistent with WHO defined radiological pneumonia [2 (2%) had primary end point consolidation, and 96 (96%) had other infiltrates], which did not differ by group. Radiographic findings were not associated with duration of illness or hospitalisation in the analysis.

**Fig 2:**
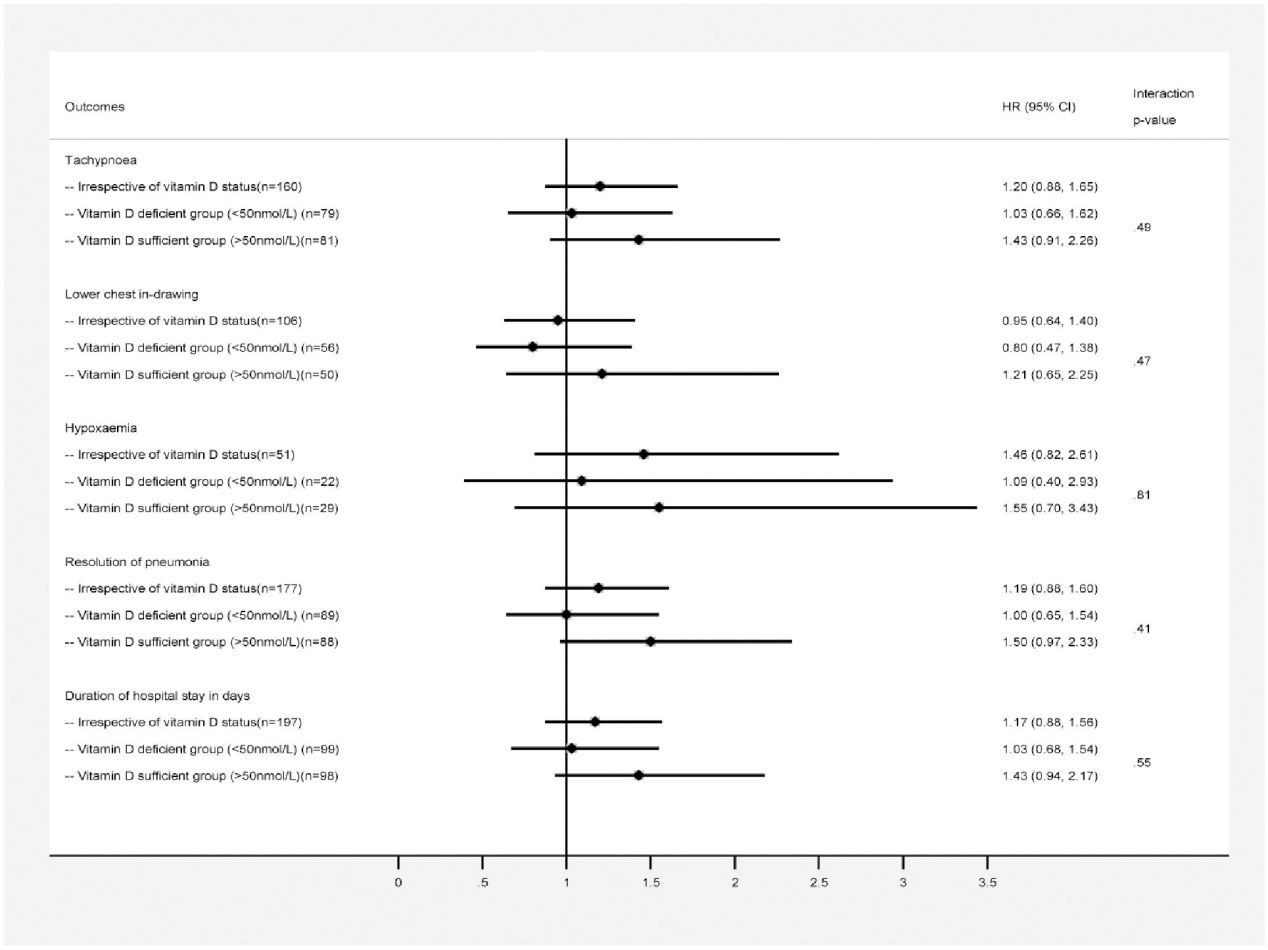
Age adjusted hazard ratios and outcome duration in hour by treatment groups. **Foot Note:** p-values are >0.05 and indicates that there is no significant interaction observed between vitamin D status (deficient/sufficient) in children and vitamin D treatment groups for each endpoint.

For each endpoint of the outcome variables we found no significant interaction between serum vitamin D status (sufficient and deficient) of the children and vitamin D treatment groups. However considering the lower trend of outcome duration in hours according to serum vitamin D sufficiency level we reported the difference with hazard ratio according to vitamin D status in three different stratums. In Fig 2, a lower trend in time to resolution of severe pneumonia (in hours) among vitamin D sufficient children was observed in vitamin D group compared to placebo [72 (IQR: 44–96) vs. 88 (IQR: 48–132); HR (95% CI): 1.50 (0.97–2.33)] with a narrow difference in significance level and although the duration of hypoxaemia (in hours) in vitamin D group was half compared to placebo but the difference was not significant [24 (IQR: 16–64) vs. 48 (IQR: 24–96); HR (95% CI): 1.55 (0.70–3.43)]. There was no difference by treatment group in time to resolution of tachypnoea, chest in-drawing, hypoxaemia or resolution of severe pneumonia irrespective of vitamin D status or vitamin D deficient status (Fig 2: Forest plot). The number of days from admission to recovery was the same for both the treatment group, irrespective of vitamin D status [Vitamin D: 4 (IQR: 3–6) vs. Placebo: 5 (IQR: 3–7); HR (95% CI): 1.17 (0.88–1.56)] and in vitamin D deficient status [Vitamin D: 5 (IQR: 4–7) vs. Placebo: 4 (IQR: 3–8); HR (95% CI): 1.03 (0.68–1.54)] (Fig 2). However, among children having sufficient vitamin D level at admission, duration of hospital stay (in days) was observed to have a lower trend in vitamin D group compared to placebo group [4 (IQR: 3–5) vs. 5 (IQR: 4–7); HR (95% CI): 1.43 (0.94–2.17)] with a narrow difference in significance.

The proportions of children with a single episode and those with multiple (>1) episodes of recurrence of pneumonia within the six month post-hospitalization follow-up period were comparable between the groups (Table 3). The risk of a “pneumonia” recurrence [vitamin D: 43 (47%); placebo: 41 (46%); risk ratio: 0.98 (95% CI 0·53–1·80)] and “severe pneumonia” [vitamin D: 12 (13%); placebo: 14 (16%); risk ratio: 0.81 (95% CI 0·35–1·87)] recurrence within 6 months of supplementation was also comparable between the two groups (Table 3).

**Table 3:** Risk of new episode of pneumonia after recovery from index episode of pneumonia during six months follow up period by treatment groups with intention to treat analysis.

Outcomes	Vitamin D group (N = 91) N (%)	Placebo group (N = 89) N (%)	Risk ratio (RR)	P-value
Children with 1 episode of cough and cold	37 (40.7)	34 (38.2)	1.38 (0.67–2.87)	0.38
Children with > 1 episode of cough and cold	32 (35.2)	27 (30.3)	1.51 (0.71–3.22)	0.29
Children with 1 episode of pneumonia	43 (47.3)	41 (46.1)	0.98 (0.53–1.8)	0.94
Children with > 1 episode of pneumonia	4 (4.4)	7 (7.9)	0.53 (0.15–1.95)	0.34
Children with 1 episode of severe pneumonia	12 (13.2)	14 (15.7)	0.81 (0.35–1.87)	0.63
Children with > 1 episode of severe pneumonia	**---**	**--**	**--**	**--**

In supplementary table, based on clinical differences in treatment groups for gender, breastfeeding, maternal education, body temperature, rhonchi, diarrhoea and dehydration (Table 1), we conducted multivariable adjusted hazard ratios for outcome median duration in hour by treatment groups. We found no difference in results for the outcome variables except for the secondary outcome variable “hypoxaemia” in the vitamin D sufficient group only [aHR (95% CI): 21.86 (1.32, 362.04)].

## Discussion

This RCT has observed clinically a trend to reductions of time to recovery and overall duration of hospital stay in children who had a sufficient serum vitamin D level on hospital admission and who received vitamin D supplementation along with standard care including antibiotic treatment. Nevertheless, our study was not adequately powered to detect the impact of vitamin D in this subset of population and thereby these findings suggest future studies in children having sufficient serum vitamin D level. However, this trial did not find any impact of adjunct vitamin D supplementation with antibiotic therapy on clinical improvement from severe pneumonia and duration of hospital stay in vitamin D deficient children and irrespective of vitamin D status of the children at baseline. Moreover, there were no differences in the risk of either a single or multiple episodes of pneumonia recurrence during 6 months follow-up period between the groups. To date, seven clinical trials have investigated potential beneficial effect of vitamin D supplementation along with standard antibiotic therapy in children admitted with acute pneumonia [25], however none have observed a benefit of vitamin D supplementation except for Rahmati et al. in 2016. Rahmati et al. reported a shorter duration of hospitalization in those who received vitamin D along with antibiotic therapy [37]. Most of these studies, including ours, were conducted in low-income countries (Afghanistan, Pakistan, and India) with a higher prevalence of malnutrition, which is associated with vitamin D deficiency and an impaired immune response.

Only two studies [26,38] including ours have included information on malnutrition, which was present in approximately 60% in both the groups in our study. Moreover, approximately 50% children in both the groups in our study were vitamin D deficient. The coexistence of malnutrition might affect the immune status and thus blunt the impact of vitamin D [39]. It has also been proposed that the systematic acute inflammatory response itself might decrease the serum vitamin D concentration through utilizing 25(OH)D [40] and we may speculate this may impede immune response in the study children despite of their receipt of vitamin D supplementation due to existing vitamin D deficiency. The observation in our study of a beneficial effect of vitamin D supplementation in children with a sufficient vitamin D level suggests the possibility of a pharmacologic effect independent of vitamin D adequacy as currently defined and warrants further research.

Not unexpectedly, vitamin D supplementation increased the serum vitamin D concentration. Despite the improvement of serum 25(OH) D levels, supplemental vitamin D did not result in an apparent therapeutic benefit of the index pneumonia episode or preventive benefit of additional episodes of pneumonia in the 6-month follow-up period, irrespective of vitamin D status or vitamin D deficiency. A possible explanation could be, only a subgroup of study children might have been benefitted from vitamin D supplementation, whereas the effect of vitamin D supplementation was obscured in children having severe vitamin D deficiency [41]. This is in contrast to the studies in Kabul and Pakistan in which vitamin D supplementation resulted in a reduction of pneumonia incidence within one and three months subsequent to hospital discharge [30,38]. The difference in this regard between these studies and our might be explained by differences in the study populations. Specifically, the current study included all the children according to WHO case definition of severe pneumonia irrespective of wheezing or nutritional status. In contrast, the Kabul study included children with pneumonia of any severity and excluded those with wheeze, because wheezing is not specific to pneumonia and can be due to bronchiolitis or an allergic respiratory condition; the study in Pakistan included children with pneumonia and excluded all the children with severe malnutrition.

Differences in vitamin D dosing have an apparent potential to influence the effect of supplementation. Daily doses of vitamin D supplementation have been observed to result in better therapeutic efficacy compared to a mega dose [29,41,42]. In our study, we provided an age-specific mega dose of vitamin D supplementation followed by a daily maintenance dose for four consecutive days. This vitamin D supplementation regimen was well tolerated and without any adverse event or clinical manifestation of vitamin D toxicity with close monitoring of the children by study physicians. As another precaution, we measured the serum ionized calcium level on study entry and administered the vitamin D or placebo only after confirmation of a calcium level within the normal range. Moreover, quantification of dietary vitamin D intake indicated no difference between the groups and therefore was unlikely to be a variable that affected the results.

Our study had certain limitations and thus it may not be justified to generalize our study findings for different settings. The main limitation of our study was more than half of our study population was severely malnourished and half of the study children were vitamin D deficient which might have a major impact on our study results through blunted immune response.

## Conclusion

Our RCT has demonstrated that an age specific mega dose of vitamin D followed by a maintenance dose have no statistical difference between the two intervention groups, however there was a trend of reduction of time to recovery from severe pneumonia and overall duration of hospital stay in under-five children with a sufficient serum vitamin D level on hospital admission. Our study findings with existing other similar studies may undermine the use of vitamin D supplement along with antibiotic therapy in children with severe pneumonia in low income countries with a high prevalence of severe malnutrition. However, our study results may be validated through further trials in well nourished children having severe pneumonia without any vitamin D deficiency in order to assess the full potential of this intervention that may help to improve outcomes of such children.

## Supporting information

S1 Checklist CONSORT 2010 checklist of information to include when reporting a randomised trial*. (DOC)

S1 Table Multivariable adjusted hazard ratios for outcome median duration in hour by treatment groups. (DOCX)

S1 File Standard management guidelines for Dhaka Hospital. (DOCX)

S2 File Vitamin D protocol. (DOC)

S3 File Vitamin D Data set. (DTA)

S4 File Vitamin D Do file. (DO)
