# Supplementary material for: Vitamin D supplementation among Bangladeshi children under-five years of age hospitalised for severe pneumonia: A randomised placebo controlled trial
Source: PLoS One. 2021 Feb 19;16(2):e0246460. doi: 10.1371/journal.pone.0246460 (PMC7894897; doi:10.1371/journal.pone.0246460)
Supplement: S1 Table — (DOCX) [file pone.0246460.s002.docx]

**Supplementary Table: Multivariable adjusted hazard ratios for outcome duration in hour by treatment groups**

| **Outcomes** | **Vitamin D**  **Median (IQR)** | **Placebo**  **Median (IQR)** | **aHR 95%CI** | **P-value** |
| --- | --- | --- | --- | --- |
| **Irrespective of vitamin D status** | | | | |
| Tachypnoea | 48 (16-96) | 48 (12-88) | 1.45 (0.86, 2.45) | 0.164 |
| Lower chest in-drawing | 64 (48-96) | 64 (40-88) | 1.02 (0.48, 2.19) | 0.953 |
| Hypoxaemia | 28 (16-56) | 40 (24-96) | 2.68 (0.89, 8.05) | 0.079 |
| Resolution of pneumonia | 72 (48-112) | 72 (48-128) | 1.36 (0.85, 2.17) | 0.198 |
| Duration of hospital stay in days | 4 (3-6) | 5 (3-7) | 1.32 (0.86, 2.03) | 0.197 |
| **Vitamin D deficient group (<50nmol/L)** | | | | |
| Tachypnoea | 48 (16-96) | 48 (24-88) | 1.31 (0.65, 2.64) | 0.449 |
| Lower chest in-drawing | 72 (48-104) | 64 (40-88) | 0.84 (0.25, 2.85) | 0.784 |
| Hypoxaemia | 40 (12-56) | 32 (24-120) | 0.21 (0.01, 4.84) | 0.331 |
| Resolution of pneumonia | 72 (64-112) | 64 (48-120) | 1.22 (0.66, 2.26) | 0.523 |
| Duration of hospital stay in days | 5 (4-7) | 4 (3-8) | 1.23 (0.70, 2.14) | 0.469 |
| **Vitamin D sufficient group (>50nmol/L)** | | | | |
| Tachypnoea | 48 (24-96) | 48 (8-96) | 2.39 (0.78, 7.31) | 0.128 |
| Lower chest in-drawing | 64 (48-96) | 60 (40-96) | 0.88 (0.11, 6.83) | 0.903 |
| Hypoxaemia | 24 (16-64) | 48 (24-96) | 21.86 (1.32, 362.04) | 0.031 |
| Resolution of pneumonia | 72 (44-96) | 88 (48-132) | 2.19 (0.87, 5.50) | 0.093 |
| Duration of hospital stay in days | 4 (3-5) | 5 (4-7) | 1.55 (0.69, 3.51) | 0.293 |

*****aHR=hazard ratio adjusted for gender, breastfeeding, maternal education, body temperature, rhonchi, diarrhoea and dehydration
