# Supplementary material for: Vitamin D supplementation among Bangladeshi children under-five years of age hospitalised for severe pneumonia: A randomised placebo controlled trial
Source: PLoS One. 2021 Feb 19;16(2):e0246460. doi: 10.1371/journal.pone.0246460 (PMC7894897; doi:10.1371/journal.pone.0246460)
Supplement: S1 File — (DOCX) [file pone.0246460.s003.docx]

**ANNEXURE**

**ANNEXURE-1-A: Voluntary Consent Form for Piloting (English)**

| Protocol No. 13088 | Version No. 1.00 | Date: 24-10-2013 |
| --- | --- | --- |

Protocol Title: Vitamin D supplementation: Impact on severe pneumonia among under-five children

Investigator’s name: Fahmida Chowdhury

Organization: icddr,b

**Purpose of the research**

**Background**

Your child is suffering from severe pneumonia, which is the leading cause of morbidity among under-five children, in developing countries including Bangladesh. Such children require admission to a hospital for treatment and care for at least five days.

There is a micronutrient known as Vitamin D. We think addition of vitamin D in the routine management of children, as stated above, may help faster recovery of children from severe pneumonia and shorten the duration of their hospital stay. However, we need to conduct a research study to determine if treatment with vitamin D could actually help earlier recovery of children with severe pneumonia and thus their earlier discharge from the hospital. We are conducting such a research study at this hospital on 350 under-five children with severe pneumonia. Before conducting the study we are intending to determine the safe dose of vitamin D intervention for the children through a pilot study. There are some studies in other countries where they have used high dose of vitamin D in children without showing any side effect. For piloting, comparing to those studies we are using two separate lower dose schedule in two groups of children to understand which dose schedule will be better for the children for the main study. For this purpose we are enrolling a total of twenty children in two groups i.e. ten children in each group. Children in each group will receive vitamin D in two different doses.

**Why invited to participate in the study?**

We need to enrol children under five years of age with severe pneumonia to test which dose of vitamin D would be safe in their treatment. As your child is suffering from severe pneumonia, we request you to help us by allowing enrolment of your child in this study.

**Methods and procedures**

If you allow participation of your child in our study, you may expect the followings:

- We would admit your child to the Respiratory Ward of this hospital, arrange for her/his appropriate treatment in accordance with guidelines of this hospital, and keep your child admitted until she/he recovers from pneumonia.
- We would ask you some questions related to your child’s illness, and perform her/his thorough physical examinations at the time of admission and on each day of hospitalisation to assess progress of illness (improvement or deterioration).
- All children, including your child, will receive the standard good treatment and care of this hospital.
- In addition to chest X-ray we will collect 5.0 mL (about one teaspoonful) of blood from a suitable

vein on your child’s arm for blood test on day of admission, 1ml of blood on 2^nd^ day, and 2 ml of blood on day of discharge and after two weeks of discharge. We have to assess vitamin D and calcium level in blood of your child through these tests to measure the safety level. We are requesting you to bring your child for follow-up after two weeks of discharge from hospital and we will collect 2.0 ml blood from your child at that time for test.

- Ten children from each group will receive vitamin D in two different doses in addition to the standard management of this hospital. Vitamin D will be administered by mixing that in expressed breast milk of the mothers/infant formula for infants younger than six months, and by mixing that in diets (such as milk suji) if the child is 6 months of age or older.

**Risk and benefits**

With the exception of receiving vitamin D, the treatment and care of the children will not differ from what would be routinely done at this hospital. We will use doses of vitamin D that is expected to be safe. We will regularly monitor your child’s clinical condition with testing serum vitamin D and calcium level. If serum vitamin D or calcium level is found to be higher than the normal range or any symptom of hypervitaminosis develops we will immediately stop further intervention so that your child may not have any adverse effect and treat accordingly. Collection of 5.0 mL of blood for this research study will not cause any harm to your child, except that she/he will feel momentary pain from the needle stick. There is rare chance of temporary discolouration of skin surrounding the needle prick, and very rare chance of infection at the prick site and/or in the blood. However, we will take necessary precautions to prevent such problems, including undertaking of sterile precautions using disposable syringes and needles. We will provide best possible treatment at this hospital, at no cost to you, in the event of any infection.

However, with the exception of more close observation by the research team and the standard treatment of this hospital, your child may not receive any other benefit from participation in this study. We are also not certain about the benefits of vitamin D in the treatment of severe pneumonia. The results of this study would improve our knowledge, and if vitamin D is proved to be beneficial that would improve management of children with severe pneumonia in the future, benefitting millions of children in Bangladesh and globally every year.

**Privacy, anonymity and confidentiality**

We do hereby affirm that privacy, anonymity and confidentiality of data/information identifying your child will strictly be maintained. We would keep all medical information, description of treatment, and results of the laboratory tests performed on your child confidential, under lock and key, and none other than our research staff will have an access to these information.

**Future use of information**

In future, information collected from you may be used by another researcher. But in that case privacy, anonymity and confidentiality of information would be maintained in such way that it would not identify you.

**Right not to participate and withdraw**

Participation of your child in this study is voluntary. Refusal to take part in the study will involve no penalty and your child will receive the standard treatment of this hospital. You have right to withdraw your child at any time from the study and you also have right to refuse answering any question.

**Principle of compensation**

Treatment at this hospital is free for all patients, and your child will not be any exception. Similarly, we will not pay money for participation of your child in our study. You will only receive the transport cost for follow up visit of your child.

We will happily provide you further information about the study, if any, now or at a later time. You may communicate with the principal investigators of the study at the contact address given below. We will answer to your question related to your patent’s medical condition, treatment, and results of any or all tests performed on your patient. However, we would like to inform you that some of the tests will be performed at the end of the study, and thus their results would be available only then.

If you agree to our proposal of enrolling your child in our study, please indicate that by putting your signature or your left thumb impression at the specified space below

Thank you for your cooperation

_______________________________________ ____________________

Signature or left thumb impression of Date

Parent/ Guardian/ Attendant

_______________________________________ ____________________

Signature or left thumb impression of the witness Date

_______________________________________ ___________________

Signature of the PI or his/her representative Date

(NOTE: In case of representative of the PI, she/he shall put her/his full name and designation and then sign)

(Name and contact phone of IRB Secretariat, RA, M. A. Salam Khan, Phone No: 9886498 or PABX 8860523-32 Extension. 3206; Principal Investigator, Dr. Fahmida Chowdhury, Phone No. PABX 8860523-32 Extension. 2550).

**ANNEXURE-2: CASE REPORT FORM (CRF)**

**Vitamin D supplementation: Impact on severe pneumonia among under-five children**

Child’s name: ………………………………………………………………………………………………

Father’s name: ……………………………………………………………………………………………..

Address: ……………………………………………………………………………………………………..

…………………………………………………………………………………………………….

Phone number: ………………………………………………………………………………………………

**Socio-demographic History:**  **Code**

1. Study ID number
2. Date of admission

3. Date of enrolment

4. Date of discharge

5. Hospital Registration No:

6. Allocation number

7. Allocation group:1=A, 2=B

# 8. Place of normal residence of the patient

(1=within Dhaka city, 2=within Dhaka district, 3= outside Dhaka district)

9. Age (in months)

10. Sex: 1=Male , 2= Female

11. Religion of the patient

(1= Muslim, 2=Hindu, 3=Christian, 4=Buddhist, 5= Others)

12. Gestational age at birth (weeks) [ code 99, if not known)

13. Father’s age (in years)

14. Mother’s age (in years)

15. Education of Patient’s Father

(1=none, 2=maktab, 3=1-5 yrs of schooling, 4=6-10 yrs, 5=10-12 yrs, 6= >12 yrs of schooling)

16. Education of Patient’s Mother

(0=none, 1=maktab, 2=1-5 yrs of schooling

3=6-10 yrs, 4=10-12 yrs, 5= >12 yrs of schooling)

17. Father’s occupation

(1=unemployed, 2= day labourer, 3=hawker, 4=petty business

5= garments worker, 6=private job, 7= govt.job, 8=driver, 9=rickshawpuller)

18. Mother’s occupation

(1=house wife, 2= garments worker, 3= day labourer,

4= private job, 5= govt. job, 6= maid servant, 7=street begger)

19. Family History of Tuberculosis

(1=yes, 2=no)

20. Family History of respiratory tract infection (within past 14days) (1=yes, 2=no)

21. Past History of pneumonia

(1=no pneumonia, 2=1 time, 3=2 times, 4=>2 times)

22. Breast feeding

(1=yes, 2=no, 9=not applicable)

23. Duration of breast feeding (in months)

24. Exclusive breast feeding

(1=yes, 2=no, 9=not applicable)

25. If non breast fed then type of feed

(1=cereal, 2=formula milk, 3=cow’s milk, 4=others)

26. Duration of complementary feeding (in months)

27. Number of rooms in the house hold

28. Number of adults sharing the same sleeping area with the child

29. Number of children sharing the same sleeping area with the child

30. Cooks inside bedroom

(1=yes, 2=no)

31. Monthly income (BDT)

32. History of smoking in the family

(1=none smokes, 2=father smokes, 3=mother smokes, 4=both parents smoke

5= other family member smokes)

33. Type of locality where care giver live?

(1=slum, 2=common housing area, 3= residential area, 4= village, 5= others)

34. Dwelling status (sun exposure)

(1=not at all, 2=mild, 3=moderate, 4=adequate)

35. How much time spends at outdoor daily (in hours)?

36. Percentage of body covered with clothes when outdoor (%)

[according to “rule of nines”- head and neck (18%), body (36%),

upper limb (18%), lower limb (28%), genitalia (1%)]

37. Number of siblings

38. Birth order of the diseased child

39. Number of under-five children in the family other than the diseased child

40. How many people live in the family?

41. Immunization status

BCG 1= Yes, 2=No , 3=Don’t know, 9=NA

Penta 1 1= Yes, 2=No, 3=Don’t know, 9=NA

Penta 2 1= Yes, 2=No, 3=Don’t know , 9=NA

Penta 3 1= Yes, 2=No, 3=Don’t know, 9=NA

Oral polio 4 1=Yes, 2=No, 3=Don’t know, 9=NA

MR 1= Yes, 2=No, 3=Don’t know, 9=NA

Measles 2 1=Yes, 2=No, 3=Don’t know, 9=NA

42. Whom did you consulted first for this episode of illness of your child?

(1= qualified doctor, 2= homeopath doctor 3= spiritual adviser

4= quack, 5= drug seller, 6=traditional healer, 7= others, 8=none)

43. Why delay (if any)?

(1= financial constraint, 2= to take care at home, 3= under treatment of quack

4= under homoeopath or spiritual treatment, 5= could not realise the problem

6= single at home, 7= others, 9=not applicable)

44. a. Use of antibiotics before admission in hospital

(1=yes, 2=no, 3=don’t know, 4=not applicable)

b. Name of antibiotic used before admission in hospital

(1=Amoxycillin, 2=Cefixime, 3=Azythromycin, 4=Ciprofloxacin, 5=Ceftriaxone

6=Cefpodoxime, 9=Don’t know)

45**. Symptoms on admission**

| **Symptoms** | **1 = yes / 2 = no** | **Duration in days** |
| --- | --- | --- |
| Diarrhoea  (Type of diarrhea:1=AWD,2=ID,3=PD) |  |  |
| Cough |  |  |
| Running nose |  |  |
| Fever |  |  |
| Vomiting |  |  |
| Respiratory distress |  |  |
| Poor oral intake |  |  |
| Lethargy |  |  |
| Convulsion |  |  |
| Others 1 |  |  |
| Others 2 |  |  |
| Others 3 |  |  |
| Others 4 |  |  |

46.**Clinical Examination**

**A) ANTHROPOMETRY**

- Admission height/length (cm)
- Admission weight (kg)
- W/A%
- ZW (z score)
- W/L%
- WL (z score)

- L/A%
- LA (z score)

**B) General examination**

- Radial pulse (rate/min) [ If imperceptible, code “000”]
- Respiratory rate (per minute)
- Axillary temperature (°C)
- Pallor

(1=yes, 2=no)

- Cyanosis

(1=yes, 2=no)

- Icterus

(1=yes, 2=no)

- Clubbing

(1=yes, 2=no)

- Pedal edema

(1=yes, 2=no)

- Lymphadenopathy

(1=yes, 2=no)

- Spo2 (%)
- Dehydration

(1=no sign, 2=some, 3=severe, 9=not applicable)

**C) Systemic examination**

1. **Respiratory system**

- Lower chest wall in drawing 1=yes, 2=no
- Nasal flaring 1=yes, 2=no

- Central cyanosis 1=yes, 2=no
- Grunting 1=yes, 2=no
- Head nodding 1=yes, 2=no
- Crackles 1=yes, 2=no
- Rhonchi 1=yes, 2=no
- Bronchial breathing 1=yes, 2=no
- Wheezing 1=yes, 2=no

1. **Cardiovascular system**

- Murmur

(1=yes, 2=no)

c**) Abdominal system**

- Liver palpable

(1=yes, 2=no)

- Spleen palpable

(1=yes, 2=no)

**d) Central nervous system**

Glasgow coma scale(GCS)

Physician’s initials: ____________ Date: ___/___/_____

47**. Laboratory investigations**

- Total WBC/cu.mm (Not done= 99)
- Poly% (Not done= 99)
- Lymphocytes (%) (Not done= 99)
- Immature poly (Band) % (Not done= 99)
- Hct% (Not done= 99)
- Sodium; mmol/L (Not done= 99)
- Potassium; mmol/L (Not done= 99)
- Chloride; mmol/L (Not done= 99)
- TCO_2_; mmol/L (Not done= 99)
- HCO3; mmol/L (Not done= 99)

- C-reactive protein on admission (Not done= 99)
- Blood glucose on admission(mmol/L) (Not done= 99)
- Serum “25 (OH)-D” level on admission(nmol/L) (Not done= 99)
- Serum “25 (OH)-D” level on discharge(nmol/L) (Not done= 99)
- Serum ionized Ca on admission(mmol/L) (Not done= 99)
- Serum ionized Ca on discharge(mmol/L) (Not done= 99)
- Serum phosphorus on admission(mmol/L) (Not done= 99)
- Serum Alkaline phosphatase on admission(U/L) (Not done= 99)
- Serum PTH on admission (pmol/L) (Not done= 99)
- Serum PTH at discharge (pmol/L) (Not done= 99)
- Blood culture 1=Positive, 2= Negative, 3=Not done

- Blood culture isolate :

(1=Streptococcus pneumoniae, 2=Staphylococcus aureus, 3=Haemophylus influenzae, 4=Salmonella typhi, 5=Salmonella para-typhi, 6=Non typhoidal Salmonella, 7=E. Coli, 8=Klebsiella, 9=Enterococcus, 10=Acinetobacter, 11=Pseudomonas,12=CNS, 13=Enterobacter species , 14=Poly microbial)

- Chest X-ray findings:

(1=normal, 2=primary end point consolidation, 3=other consolidation/infiltrate,

4=pleural effusion, 5=pneumothorax)

48**. Diet with Vitamin D3: / Diet without Vitamin D3:**

| Day | Date | Time | Dose given | If any vomiting within 30 mins, repeat the dose | If any adverse effect* |
| --- | --- | --- | --- | --- | --- |
| 1. |  |  |  |  |  |
| 2. |  |  |  |  |  |
| 3. |  |  |  |  |  |
| 4. |  |  |  |  |  |
| 5. |  |  |  |  |  |

(*Adverse effect: vomiting, decreased appetite, irritability, constipation, dehydration, fatigue, muscle weakness)

49. **Antibiotics given:**

| Antibiotics | 1=yes,2=no | Day started | Day stopped | Comments |
| --- | --- | --- | --- | --- |
| Ampicilin |  |  |  |  |
| Gentamicin |  |  |  |  |
| Ceftriaxone |  |  |  |  |
| Levofloxacin |  |  |  |  |
| Ceftazidime |  |  |  |  |
| Amikacin |  |  |  |  |
| Flucloxacillin |  |  |  |  |
| Vancomycin |  |  |  |  |
| Imipenem |  |  |  |  |
| Meropenem |  |  |  |  |
| Cotrimoxazole |  |  |  |  |
| Ciprofloxacin |  |  |  |  |
| Clarithromycin |  |  |  |  |

50. The amount of vitamin D received through dietary source (IU)

(Infant formula/milk siji)

51. Final Outcome

- - - 1. Well & discharged
      2. Did not improve but discharged on request
      3. Died in hospital
      4. DORB
      5. Absconded
      6. Referred to another hospital

52. Duration of hospitalization (in days)

Physician’s initials: ___________________ Date: ___/___/____

**Follow up During Hospital Stay**

| Days | Date | Time | Pulse/min | Resp rate  /min | Chest indrawing | Spo2  (%) | | Temp  (◦C) | Lungs  (1=fine creps  2=coarse creps  3=clear) | Cough | Feeding  (1=well 2=poor) | Lethargy | Convulsion | AWD | Sign symtomps of Raised ICP | Pneumonia status^*^ |
| --- | --- | --- | --- | --- | --- | --- | --- | --- | --- | --- | --- | --- | --- | --- | --- | --- |
| **Day 1** |  |  |  |  |  |  |  | |  |  |  |  |  |  |  |  |
| 0 hrs |  |  |  |  |  |  |  | |  |  |  |  |  |  |  |  |
| 8 hrs |  |  |  |  |  |  |  | |  |  |  |  |  |  |  |  |
| 16 hrs |  |  |  |  |  |  |  | |  |  |  |  |  |  |  |  |
| 24 hrs |  |  |  |  |  |  |  | |  |  |  |  |  |  |  |  |
| **Day 2** |  |  |  |  |  |  |  | |  |  |  |  |  |  |  |  |
| 8hrs |  |  |  |  |  |  |  | |  |  |  |  |  |  |  |  |
| 16 hrs |  |  |  |  |  |  |  | |  |  |  |  |  |  |  |  |
| 24 hrs |  |  |  |  |  |  |  | |  |  |  |  |  |  |  |  |
| **Day 3** |  |  |  |  |  |  |  | |  |  |  |  |  |  |  |  |
| 8 hrs |  |  |  |  |  |  |  | |  |  |  |  |  |  |  |  |
| 16 hrs |  |  |  |  |  |  |  | |  |  |  |  |  |  |  |  |
| 24 hrs |  |  |  |  |  |  |  | |  |  |  |  |  |  |  |  |
| **Day 4** |  |  |  |  |  |  |  | |  |  |  |  |  |  |  |  |
| 8hrs |  |  |  |  |  |  |  | |  |  |  |  |  |  |  |  |
| 16 hrs |  |  |  |  |  |  |  | |  |  |  |  |  |  |  |  |
| 24 hrs |  |  |  |  |  |  |  | |  |  |  |  |  |  |  |  |
| **Day 5** |  |  |  |  |  |  |  | |  |  |  |  |  |  |  |  |
| 8hrs |  |  |  |  |  |  |  | |  |  |  |  |  |  |  |  |
| 16hrs |  |  |  |  |  |  |  | |  |  |  |  |  |  |  |  |
| 24 hrs |  |  |  |  |  |  |  | |  |  |  |  |  |  |  |  |

(Pneumonia status^*^ 1=severe pneumonia continues, 2=severe pneumonia resolved, 3=pneumonia continues, 4=pneumonia resolved)

| Days | Date | Time | Pulse/min | Resp rate/min | Chest indrawing | Spo2  (%) | Temp  (◦C) | Lungs  (1=fine creps  2=coarse creps  3=clear) | Cough | Feeding  (1=well 2=poor) | Lethargy | Convulsion | AWD | Sign symtomps of Raised ICP | Pneumoia status* |
| --- | --- | --- | --- | --- | --- | --- | --- | --- | --- | --- | --- | --- | --- | --- | --- |
| **Day 6** |  |  |  |  |  |  |  |  |  |  |  |  |  |  |  |
| 0 hrs |  |  |  |  |  |  |  |  |  |  |  |  |  |  |  |
| 8 hrs |  |  |  |  |  |  |  |  |  |  |  |  |  |  |  |
| 16 hrs |  |  |  |  |  |  |  |  |  |  |  |  |  |  |  |
| 24 hrs |  |  |  |  |  |  |  |  |  |  |  |  |  |  |  |
| **Day 7** |  |  |  |  |  |  |  |  |  |  |  |  |  |  |  |
| 8hrs |  |  |  |  |  |  |  |  |  |  |  |  |  |  |  |
| 16 hrs |  |  |  |  |  |  |  |  |  |  |  |  |  |  |  |
| 24 hrs |  |  |  |  |  |  |  |  |  |  |  |  |  |  |  |
| **Day 8** |  |  |  |  |  |  |  |  |  |  |  |  |  |  |  |
| 8 hrs |  |  |  |  |  |  |  |  |  |  |  |  |  |  |  |
| 16 hrs |  |  |  |  |  |  |  |  |  |  |  |  |  |  |  |
| 24 hrs |  |  |  |  |  |  |  |  |  |  |  |  |  |  |  |
| **Day 9** |  |  |  |  |  |  |  |  |  |  |  |  |  |  |  |
| 8hrs |  |  |  |  |  |  |  |  |  |  |  |  |  |  |  |
| 16 hrs |  |  |  |  |  |  |  |  |  |  |  |  |  |  |  |
| 24 hrs |  |  |  |  |  |  |  |  |  |  |  |  |  |  |  |
| **Day10** |  |  |  |  |  |  |  |  |  |  |  |  |  |  |  |
| 8hrs |  |  |  |  |  |  |  |  |  |  |  |  |  |  |  |
| 16hrs |  |  |  |  |  |  |  |  |  |  |  |  |  |  |  |
| 24 hrs |  |  |  |  |  |  |  |  |  |  |  |  |  |  |  |

(Pneumonia status^*^ 1=severe pneumonia continues, 2=severe pneumonia resolved, 3=pneumonia continues, 4=pneumonia resolved)

**Follow up at home after discharge**

| **Weeks** | **Date** | **No ARI illness** | **No pneumonia (cough and cold)** | **Pneumonia** | **Severe pneumonia** | **Other illness** | **Final Outcome** |
| --- | --- | --- | --- | --- | --- | --- | --- |
| 1^st^ week |  |  |  |  |  |  |  |
| 2^nd^ week |  |  |  |  |  |  |  |
| 3^rd^ week |  |  |  |  |  |  |  |
| 4^th^ week |  |  |  |  |  |  |  |
| 5^th^ week |  |  |  |  |  |  |  |
| 6^th^ week |  |  |  |  |  |  |  |
| 7^th^ week |  |  |  |  |  |  |  |
| 8^th^ week |  |  |  |  |  |  |  |
| 9^th^ week |  |  |  |  |  |  |  |
| 10^th^ week |  |  |  |  |  |  |  |
| 11^th^ week |  |  |  |  |  |  |  |
| 12^th^ week |  |  |  |  |  |  |  |
| 13^th^ week |  |  |  |  |  |  |  |
| 14^th^ week |  |  |  |  |  |  |  |
| 15^th^ week |  |  |  |  |  |  |  |
| 16^th^ week |  |  |  |  |  |  |  |
| 17^th^ week |  |  |  |  |  |  |  |
| 18^th^ week |  |  |  |  |  |  |  |
| 19^th^ week |  |  |  |  |  |  |  |
| 20^th^ week |  |  |  |  |  |  |  |
| 21^st^ week |  |  |  |  |  |  |  |
| 22^nd^ week |  |  |  |  |  |  |  |
| 23^rd^ week |  |  |  |  |  |  |  |
| 24^th^ week |  |  |  |  |  |  |  |

**Standard Management Guidelines for Dhaka Hospital (Annexure 5)**
