# Supplementary material for: Vitamin D supplementation among Bangladeshi children under-five years of age hospitalised for severe pneumonia: A randomised placebo controlled trial
Source: PLoS One. 2021 Feb 19;16(2):e0246460. doi: 10.1371/journal.pone.0246460 (PMC7894897; doi:10.1371/journal.pone.0246460)
Supplement: S2 File — (DOC) [file pone.0246460.s004.doc]

**Protocol Title:** Vitamin D supplementation: Impact on severe pneumonia among under-five children

| Project Summary |
| --- |
| Principal Investigator: Fahmida Chowdhury |
| Research Protocol Title: Vitamin D supplementation: Impact on severe pneumonia among under-five children |
| **Background:**   1. Burden: Pneumonia is the leading cause of morbidity and mortality in under-five children, particularly in developing countries. 2. Knowledge gap: Although many studies have reported an association between vitamin D deficiency and pneumonia, there is lack in information on its therapeutic impact, i.e. the impact of vitamin D supplementation in the management of childhood pneumonia. 3. Relevance: Vitamin D plays an important role in modulating the innate immune response against infections. We, therefore, propose to conduct this study to assess the impact of vitamin D supplementation, in addition to standard antibiotic and supportive therapy, on the outcome of severe childhood pneumonia.   **Hypothesis**: We hypothesise that in the management of hospitalized severe pneumonia in under-five children, vitamin D_3_ supplementation, as an adjunct to the standard antibiotic and other supportive therapy, will hasten recover from severe pneumonia and may thereby shorten duration of severe pneumonia and also reduce the risk of new episode of pneumonia.  **Objectives:** The objective of our study is to assess the clinical benefit of oral supplementation of vitamin D_3_, in addition to standard antibiotic and other supportive therapy, to hospitalised, under-five children with severe pneumonia.  **Methods:** This would be a randomised, double blind, controlled clinical trial (RCT). Children of either sex, aged 3-59 months, attending the Dhaka Hospital of icddr,b, with clinically diagnosed severe pneumonia will comprise the study population. Eligible children will be allotted a sequential study number, which will have been previously assigned to vitamin D or placebo in accordance with the randomisation. The study staff and mothers/ caregivers of the children will be blinded as to whether vitamin D3 or placebo has been added to their child's diet. Infants aged 3-5 months will receive breast milk and/or infant formula, and those 6 months or older will receive “Milk Suji” as a complementary food. Vitamin D3 supplementation will be given on five consecutive days, from the day of enrolment in addition to standard antibiotic and other supportive therapy.  **Outcome measures/variables:**  Primary outcome measure will be time to resolution of severe pneumonia.  Secondary outcome measures will be duration of hospitalization, fever, tachypnoea, chest in drawing, hypoxia, lethargy and inability to feed during hospital stay and as well as new episode of pneumonia after discharge. |

#

**Description of the Research Project**

##

## Hypothesis to be tested:

| In a hypothesis testing research proposal, briefly mention the hypothesis to be tested and provide the scientific basis of the hypothesis, critically examining the observations leading to the formulation of the hypothesis. |
| --- |

Does this research proposal involve testing of hypothesis:  No  Yes (describe below)

We hypothesise that supplementation of vitamin D_3_ (Cholecalciferol), in addition to standard antibiotic and other supportive therapy in the management of under-five children with severe pneumonia will significantly shorten the duration of severity of pneumonia and thereby reduce hospital stay and also reduce the risk of new episode of pneumonia.

## Specific Objectives:

| Describe the specific objectives of the proposed study. State the specific parameters, gender aspects, biological functions, rates, and processes that will be assessed by specific methods. |
| --- |

**Objective:** To assess the clinical benefit of oral vitamin D_3_ supplementation, in addition to standard antibiotic and other supportive therapy, in the management of hospitalised, under-five children with severe pneumonia.

**Primary objective:** To compare the duration of severe pneumonia of children in the two study groups receiving the standard therapy (appropriate antibiotic and other supportive therapy) with those receiving vitamin D supplementation in addition to the standard therapy.

**Secondary objectives:**

- To compare the duration of hospitalization, fever, tachypnoea, chest in drawing, hypoxia, lethargy and inability to feed during hospital stay, in under-five children in the two study groups, as mentioned under the primary aim.
- To compare the recurrence of new episode of pneumonia over the next 6 months after discharge from hospital in the two study groups.
- To identify the optimum safety dose of vitamin D3 for different groups of under-five children.
- In severe pneumonia patient who also have acute watery diarrhoea we will assess the indirect absorption of vitamin D by measuring serum vitamin D level and will also assess the clinical outcome in the subgroup.

## Background of the Project including Preliminary Observations:

| Provide scientific validity of the hypothesis based on background information of the proposed study and discuss previous works on the research topic, including information on sex, gender and diversity (ethnicity, SES) by citing specific references. Critically analyze available knowledge and discuss the questions and gaps in the knowledge that need to be filled to achieve the proposed aims. If there is no sufficient information on the subject, indicate the need to develop new knowledge. |
| --- |

##

Pneumonia is the leading cause of morbidity and mortality in under-five children, particularly those living in developing countries.^1,2^ In 2010 there were 120 million episodes of pneumonia in children younger than five years and 14 million of pneumonia cases progress to severe and life-threatening condition requiring urgent hospital care.^3^ In 2011 it was estimated that 1.3 million pneumonia led to death worldwide.^3^ In Bangladesh, pneumonia remains as a significant health problem and is one of the major causes of childhood mortality.^4^ The Millennium Development Goal 4 (MDG4) aims to reduce child mortality by two thirds (2/3rds) by the year 2015. The success of achieving the target depends on reducing the prevalence of pneumonia in children along with efficient management of pneumonic children.

Many research studies have been conducted to identify effective interventions to reduce global burden of deaths due to pneumonia. They mostly focused on preventive strategies such as improving nutritional status and exclusive breast-feeding, reducing indoor air-pollution and overcrowding, improving access to effective antibiotics, care-seeking behaviour, referral practices and quality of case management.^5^  There is increasing recognition that nutritional deficiencies, including that of micronutrients, play an important role in infectious diseases, such as pneumonia and their outcome.^6^  Identifying an effective nutritional intervention, including micronutrients to reduce morbidity and shorten hospitalisation, would improve medical care and morbidity.^7^ Many trials have investigated the benefits of vitamin A supplements; however, with the exception of reduction in measles associated deaths and overall mortality, no significant reduction of morbidity and mortality from acute lower respiratory tract infections (ALRI) was observed.^8^ Zinc supplementation has been reported to reduce ALRI incidence by 41%, and has also been reported to accelerate recovery from pneumonia when used as an adjunct to effective antibiotic therapy; however, another study in India reported negative outcomes.^9-11^

Vitamin D plays a crucial role in calcium and phosphorus homeostasis, and is a key factor in skeletal mineralization. Its deficiency causes rickets and impaired skeletal growth in children and osteomalacia in adults.^12^ Clinical and subclinical vitamin D deficiency in children has been reported to be a significant risk factor for severe ALRI.^13^ In addition to maintenance of calcium levels, Vitamin D also plays an important role in modulating the innate immune response against infections.^14^

It has been reported that 1,25 dihydroxyvitamin D_3_, the active metabolite of vitamin D, is important for promoting and regulating immune responses in vitro, which is also supported by other studies in human.^12,15-17^ Scientists at the University of **Copenhagen have discovered that** when a T cell is exposed to a foreign pathogen, a signaling device or 'antenna', known as a vitamin D receptor, is extended, and it searches for vitamin D with it. This means that the T cell activation will cease without vitamin D. T cell won't even begin to mobilise if they cannot find enough vitamin D in the blood. Successfully activated T cells transform into one of two types of immune cells- they may either become killer cells that attack and destroy all cells carrying traces of foreign pathogen or may become helper for T cells to detect and kill foreign pathogens such as clumps of bacteria or viruses. T cells rely on vitamin D for their activation and they would remain dormant, ‘naïve’ to the possibility of threat if vitamin D was lacking in the blood.^18^

Research studies conducted thus far highlights the link between Vitamin D and pneumonia. For example, an Indian study reported subclinical vitamin D deficiency as a significant risk factor for severe ALRI in under-five children.^12^ An Ethiopian study reported clinical signs of rickets in 41% of 300 consecutive under-five children attending the outpatient ward; in the inpatient case control study rickets was associated with 13-fold higher risk of pneumonia.^19^ Another study in Yemen reported significant association between rickets and the treatment outcomes from very severe pneumonia.^20^ In Iran, 43% of 200 children with rickets had radiological pneumonia, and in Kuwait pneumonia was diagnosed in 44% of 250 children with Vitamin D deficiency rickets.^21,22^ A study identified 131patients between 1979 to 1988, and compared two age matched and sex matched groups- one with rickets and the other group of healthy controls without rickets. The study observed pneumonia to be more common in rachitic patients than in the controls.^23^ A hospital-based, case control study in Egypt reported ARI in 81% of children with rickets compared to 58% in the controls.^24^ A systematic review and meta-analysis of randomized controlled trials on vitamin D and respiratory tract infections reported that vitamin D has a protective effect against respiratory tract infections and dosing once daily seems most effective than bolus dose.^25^

A hospital-based, randomised, controlled trial in Kabul shows that supplementation of a bolus dose of 100,000 IU vitamin D in addition to antibiotic to young children with pneumonia was associated with reduced repeat episode of pneumonia, without reducing the hospital stay.^26^ Another study in India reported no beneficial effect of small consecutive dose of vitamin D with antibiotics on resolution of severe pneumonia among under five children.^27^

However limited information is available in Bangladesh about the vitamin D status in children. A survey conducted at Dhaka neighborhood found that among 319 children between the age group of 16 to 20 months and 164 children between the age group of 30-36 months respectively 51% and 43% had blood vitamin D (25-OH-D) level below the cut off value (47.7 nmol/L) of 25-OH-D (Personal communication from Nusrat Homaira, unpublished). From the data on National Rickets Survey in Bangladesh in 2008, it was documented that 98% children were vitamin D deficient out of 154 rachitic children (Personal communication from Dr. S k Roy, unpublished) and a study from Chakaria observed that 2 (20%) children out of 10 rachitic children were vitamin D deficient.^28^ A matched case-control study in North-Eastern part of Bangladesh noted a relationship between Vitamin D status and early childhood ALRI, and young age group was the only risk factor for vitamin D deficiency.^29^

Although many studies have shown the association between vitamin D deficiency and pneumonia, there is limited information on the effect of vitamin D supplementation in the outcome of children with pneumonia.^12, 19-27^ We are, therefore, interested to conduct a randomised, controlled clinical trial to assess the impact of vitamin D supplementation, in addition to standard antibiotic therapy, on the duration of hospitalization, as well as on the duration of severe pneumonia of the affected children and further new episode of pneumonia.

Moreover co-morbidity of pneumonia and diarrhea is a burning public health problems accounting more than one third of 7.6 million global childhoods under five deaths^30,31^ and evaluation of the role of vitamin D in such population may have great value.

## Research Design and Methods

| Describe the research design and methods and procedures to be used in achieving the specific aims of the research project. If applicable, mention the type of personal protective equipment (PPE), use of aerosol confinement, and the need for the use BSL2 or BSL3 laboratory for different part of the intended research in the methods.. Define the study population with inclusion and exclusion criteria, the sampling design, list the important outcome and exposure variables, describe the data collection methods/tools, and include any follow-up plans if applicable. Justify the scientific validity of the methodological approach (biomedical, social, gender, or environmental).  Also, discuss the limitations and difficulties of the proposed procedures and sufficiently justify the use of them. |
| --- |

**Study site:**

The study will be conducted at the Dhaka Hospital of the International Centre for Diarrhoeal Disease Research, Bangladesh (icddr,b), Dhaka, Bangladesh. This hospital has separate ward for management of children with severe pneumonia and also has an Intensive Care Unit (ICU), equipped with mechanical ventilator, cardiac monitors, and other supportive facilities for management of critically ill patients.

**Study population:**

Children of either sex, aged 3-59 months, attending the Dhaka Hospital of icddr,b, Dhaka with clinically diagnosed severe pneumonia (case definition described in a later section) will comprise the study population.

**Study design:** This would be a randomised, double blind, controlled clinical trial (RCT).

**Inclusion criteria:**

Children of either sex aged 3 - 59 months, with a clinical diagnosis of severe pneumonia with or without diarrhea.

**Exclusion criteria**

1. Known case of hypercalcaemia or allergy to vitamin D, as determined by history or previous medical records.
2. Congenital Heart disease, evidenced by clinical exam or past medical records.
3. Renal or hepatic insufficiency, evidenced by clinical exams or past medical records.
4. Known case of tuberculosis, evidenced by medical records
5. Known case of asthma, evidenced by history and clinical exam findings.
6. Critically ill children requiring ICU care, such as those with septic shock or cardiac arrest or apnoea.
7. Received vitamin D or calcium supplementation within the last 4 weeks before current admission, as evidenced by history or medical prescription.
8. Baseline ionized calcium level above the normal limit for the main phase of the study.

**Study Intervention**

Eligible children satisfying all the study criteria will be randomized, in equal numbers to receive the vitamin D3 supplementation or placebo in their diet in addition to the standard therapy (appropriate antibiotic and supportive therapy). Infants aged 3-5 months will receive breast milk and/or infant formula, and those 6 months or older will receive complementary food such as “Milk Suji”. Vitamin D3 or placebo will be administered adding with their diet. For exclusively breast fed babies vitamin D3/placebo will be administered in expressed breast milk.

We will decide the dose of vitamin D3 supplement for this study after piloting. Two studies have evaluated the impact of vitamin D3 in childhood pneumonia; one in Kabul with a bolus dose of vitamin D3 (100,000 IU) ^26^ and another study in India with small dose (1000/2000IU) ^27^ of vitamin D3 on 5 consecutive days.  Both the studies have shown no effect of vitamin D3 in hospitalized pneumonia children but the study in Kabul showed vitamin D3 supplementation could reduce the occurrence of new episode of pneumonia. There was a critical review of the second study in India which recommended use of 10,000 IU vitamin D3 on 5 consecutive days.^32^ These two studies have not done serum 25(OH)D and calcium level assay and did not find any symptoms of vitamin D toxicity or adverse effect. Although researchers first identified the fat-soluble vitamin cholecalciferol almost a century ago and studies have now largely elucidated the transcriptional mechanism of action of its hormonal form,1_,25-dihydroxyvitamin D3 [1_,25(OH)2D3], we know surprisingly little about mechanisms of vitamin D toxicity.^33^ In spite of current data support the viewpoint that the biomarker plasma 25(OH)D concentration must rise above 750 nmol/L to produce vitamin D toxicity, the more prudent upper limit of 250 nmol/L might be retained to ensure a wide safety margin.^33,34^ Therefore for this study to ensure the safe dose of vitamin D3 for the children we will first proceed through piloting.

For piloting we will enroll 20 patients and group them into two that is ten patients in each group. We will intervene one group with vitamin D3 supplement 10,000 IU for five consecutive days. Another group will receive stratified dose of vitamin D3 according to age. That is 20,000IU vitamin D3 in children <6 months, 50,000 IU in children 6-12 months and 100,000 IU in children 13-59 months of age on first day and thereafter 10,000 IU for next 4 days. We will observe any adverse event after the intervention and monitor the serum 25(OH)D level on admission, at discharge (day 5) and after two weeks of discharge as the half life of vitamin D3 is two weeks. To collect blood sample after two weeks of discharge parent/guardian will be requested for follow up visit at hospital with the child at that time. In case of failure to visit the hospital study staff will visit their home for follow up of the child and collection of blood sample for serum 25(OH)D level assay. We will also monitor serum calcium level on day of admission, 2^nd^ day and at discharge for safety monitoring as vitamin Ds primary roll is to control the levels of calcium in the body by constantly adding calcium to or taking calcium from bones. Depending on the adverse event and serum 25 (OH)D and calcium level of this pilot phase, we will decide the final dose for vitamin D3 intervention for the study. We will consider the toxic level of 25(OH)D as 250nmol/L^33,34^ and the upper limit of the serum calcium would be 2.63 mmol/L for safety monitoring.^35^ If we find serum 25(OH)D and calcium level on admission above this level we will stop further intervention. After finalization of dose in this study we will assay the 25(OH)D and calcium level on admission and on 5^th^ day or earlier at discharge.

For the study, participants will be randomly assigned to one of two masked parallel intervention groups, with allocation concealment: vitamin D3 (cholecalciferol) or matched placebo. The vitamin D3 will be a high- concentration (20,000 IU D3 per mL) liquid formulation (Vigantol Oil, Merck KGaA, Germany), and the placebo will be miglyol oil 812 (Sasol, Germany), the vehicle used in Vigantol Oil. These active supplement and selected placebo are identical in appearance and both are tasteless. We have added vigantol oil and miglyol oil in 50 ml of “Milk Suji”, infant formula and expressed breast milk and there was no difference in appearance, color, odor or taste of diets with Vigantol oil (vitamin D3) or miglyol oil (placebo).

The allocated intervention, vitamin D3/placebo will be administered by adding in the diets within two hours of the first dose of parenteral antibiotics on the day of admission after confirming baseline ionized calcium within normal range and in the first morning diet for days 2-5 of the hospitalisation. Children who are unable to take oral diet, in that case vitamin D3/placebo will be administered through nasogastric tube with their diet. Intervention will be repeated if the child has vomiting within 30 minutes of intervention.

**Vitamin D dosing for the main phase of then study:**

Based on the findings from the pilot phase of the study we have received IRB approval for the optimal safe dose of vitamin D where patient will receive stratified dose of vitamin D3 according to age. That is 20,000IU vitamin D3 in children <6 months, 50,000 IU in children 6-12 months and 100,000 IU in children 13-59 months of age on first day and thereafter 10,000 IU for next 4 days.

**Randomisation and Allocation Concealment**

The eligible children will be allotted a sequential study number, which will have been previously assigned to vitamin D3 or placebo in accordance with the randomisation. A computer generated random list will be developed by a person not involved in the study in any way. Participants and the research staff will be blinded to allocation. Pharmaceuticals Company will prepare the supplements off-site using individual opaque glass vials labeled with concealed unique identifiers for vitamin D3 and placebo. Allocation concealment will be done by sealed envelope and will be kept in a secured place by the person not associated with the study in any way; the envelope will be opened at the end of the study only, prior to data analysis.

**Definition of severe pneumonia**

Children aged 3 months to 59 months will be diagnosed as a case of severe pneumonia if they have a history of cough and/or respiratory difficulty plus oxygen saturation < 90% or central cyanosis, or severe respiratory distress (grunting, very severe chest in-drawing), or signs of pneumonia with a general danger sign (inability to breastfeed or drink, lethargy or reduced level of consciousness, convulsions), auscultatory findings of decreased or bronchial breath sounds or signs of pleural effusion or empyema.^36^

If a child presents with severe malnutrition (children with pitting oedema or Z scores below -3 standard deviations (SD) from the median for weight for height or weight for age or height for age) with any sign of pneumonia (any of the WHO defined signs of pneumonia or severe pneumonia or radiological pneumonia) would be considered as severe pneumonia.^37^

**Collection of Baseline Information**

All children within the defined age group suspected to have ALRI will be screened for study eligibility. Parents/ attending care givers of those fulfilling the eligibility, in application of the inclusion and the exclusion criteria, will be invited to provide their consent for enrollment of their children in the study. Upon signing a written informed consent, after providing information about the study and its interventions, possible benefits and risks, and voluntary nature of participation along with the right to withdraw children at any time after the initial consent without providing any reason, children will be enrolled. Case Record Form (CRF, ANNEXURE 1) will be used to collect relevant information such as medical history including nature and duration of illness, medication for current illness; socio-demographic characteristics such as sex, religion, gestational age, parental age with education, parents occupation, fuel use and smoking history, monthly family income, number of siblings, number of rooms, and cooking in bed room. Information will also be collected about child’s feeding practice such as history of breast feeding, formula or other complementary feeding, and immunisation status; family history of tuberculosis, recent respiratory tract infection of any family members and past history of child’s pneumonia, and history of exposure of the child to sun would be recorded. Medical examination findings to be recorded include pulse and respiratory rate, axillary temperature, anthropometric measurement, chest auscultation findings, chest wall in drawing, oxygen saturation, presence of cyanosis, and mental status (normal, irritable, lethargic). Weight of each child will be measured by electronic weighing scale with a precision of 0.1 kg and height/length will be measured using a locally made length board with precision of 0.1cm. Fever will be defined when the axillary temperature is 38°C or greater. Respiratory rate will be counted for full 60 seconds by exposing the trunk when the child is awake and calm, and presence of lower chest wall in drawing will be noted at the same time. Respiratory rate will be counted for two times and if they differ by more than 5 breaths per minute then a third reading will be made, and the average of two closest respiratory rates (not deviating by 5 or greater number) will be regarded as the actual rate. Oxygen saturation will be measured using a Pulse Oxymeter (Nellcor Puritan Bennett Inc. N-560, Made in Korea) with a probe on a finger or toe when the child breathes in room air. Oxygen saturation of 90% or lesser will be defined as hypoxia which will be the indication for oxygen therapy. Chest X-ray will be done after enrollment and at discharge; however, it may be done at other times if clinically indicated. Five milliliter (5.0 mL) of venous blood will be collected from the children after enrollment in the study for estimation of complete blood count, C reactive protein, blood glucose, electrolytes,

“25 (OH)D” level, Ca, phosphorus, alkaline phosphatase, as well as blood culture for bacterial pathogen before the introduction of IV antibiotics and 2 ml venous blood will be collected at discharge and after 2 weeks of discharge for serum “25 (OH)D” and Ca level assay.

We will also monitor parathormone level randomly every fifth patients on admission and at discharge during the main phase of the study.

**Monitor ionized calcium level instead of total serum calcium:**

We will do blood test for serum ionized calcium instead of total serum calcium for the main phase of the study. In our pilot phase of the study we have found about 50% of our enrolled children with severe pneumonia were severely malnourished. These children may have low albumin level and so may not reflect the actual calcium status of the body through total serum calcium. Moreover ionized calcium gives more accurate picture of calcium in blood comparing to total serum calcium.

In that case as we have considered the upper normal limit of total serum calcium for safety monitoring, in case of ionized calcium the upper limit (normal range: 1.15-1.33 mmol/L)^38^ will be considered for safety monitoring. So the child having ionized calcium above 1.33mmol/L on admission will be stopped from having further intervention and excluded from the study.

**Treatment of Severe Pneumonia**

The enrolled children will be admitted to the Respiratory Ward of the Dhaka Hospital and placed on bed. Patency of their airway will be assessed and naso-and/or oropharyngeal suction will be given to those requiring them. Oxygen will be administered to those with an oxygenation saturation of 90% or lesser while breathing in room atmosphere. After the initial collection of a blood sample children will be treated according to pneumonia treatment guidelines of the Dhaka Hospital (Community Acquired Pneumonia in Children, Document No. PR/TG/006/00) added in Annexure 4.

**Monitoring during hospital stay**

#### During the 5 days of hospital stay data will be recorded 8 hourly including pulse and respiratory rates, axillary temperature, chest auscultatory findings, presence/absence of lower chest wall in drawing, oxygen saturation, and history of cough and feeding (Annexure 3). Resolution of sever pneumonia will be defined as absence of all signs of severe pneumonia, such as lower chest wall in drawing, cyanosis, hypoxia or lethargy, convulsion and inability to feed. Resolution of pneumonia will be considered when there is no fever, tachypnoea (RR less than 40 in children above 1 year and RR less than 50 in children below 1 year) for at least 24 hours and the child can be orally fed.

We will also monitor any adverse events suspected for vitamin D3 during hospital stay. Vitamin D3 at normal doses usually has no side effects. Any sign symptoms of vitamin D toxicity such as vomiting, decreased appetite, irritability, constipation, dehydration, fatigue, muscle weakness^39^  will be monitored and if recorded will be evaluated for hypercalcaemia. If hypercalcaemia develops we will stop further intervention with vitamin D/placebo. Any deterioration of child’s condition such as septic shock, apnoea (cease of respiration for 10-15 seconds) or cardiac arrest requiring the support of bubble CPAP or mechanical ventilation at any time of the study the child will be transferred to ICU and stop further intervention with vitamin D/placebo. And they will receive the standard hospital care and will be reported to ERC (Ethical Review Committee)/DSMB (data safety and monitoring board) as a case of SAE (serious adverse event). Proportion of such children in the two study groups will be compared during data analysis. We will assess serum “25 (OH)D” and calcium level on admission. If calcium is above the safety level as mentioned above we will discontinue further intervention and exclude the child from the study.

We will also measure the amount of vitamin D patient received through dietary source (infant formula/milk suji) by calculating the total amount of milk received during hospital stay and there by the amount of vitamin D in International Unit (IU) in that amount of diet.

**Criteria for Discharge**

- Resolution of pneumonia (defined above)
- No co-morbidities requiring hospitalised treatment
- Can be put on oral antibiotic (after resolution of pneumonia)

**Follow up:**

Health worker will follow up the study children on a weekly basis over cell phone up to 6 months after discharge. They will be trained on diagnosis of acute respiratory illness according to IMCI (Integrated Management of Childhood Illness) guideline. They will ask the caregiver/mother for any symptoms of new episode of acute respiratory illness. Study children will be given an ID card to facilitate the follow up at home and revisit to the hospital if any illness develop. Follow up will be recorded in a case record form for 24 weeks (6 months) categorized as no acute respiratory illness, no pneumonia (cough and cold), pneumonia, severe pneumonia and other illness. A new episode of pneumonia will be diagnosed if an episode of pneumonia 14 days after the last day of illness of the previous episode of pneumonia occurs.

**SNP analysis:**

DNA sequence variations occur when a single nucleotide (A, T, C, or G) in the genome sequence is altered. A Single nucleotide polymorphism or SNP is a DNA sequence variation occurring when a single nucleotide - A, T, C, or G - in the genome (or other shared sequence) differs between members of a species (or between paired chromosomes in an individual).Each individual has many single nucleotide polymorphisms that together create a unique DNA pattern for that person. Variations in the DNA sequences of humans can affect how humans develop diseases, respond to pathogens, chemicals, drugs, etc.

Some of the children may have genetic polymorphism and so there may be genetic predisposition to disorders with Vitamin D metabolism.

Therefore we will store a blood sample for SNP analysis in future from the study children. We will only look for common polymorphisms, present in 10-20% of individuals, as the sample size is too small to have power for less common polymorphisms.

**DNA preparation for genome-wide association**

We will store blood sample for future DNA extraction from whole blood collected for genome wide association (GWA) from all cases. One million single nucleotide polymorphisms (SNPs) will be genotyped per individual. The goal will be to test if GWA can be used to identify genetic polymorphisms associated with vitamin D deficiency.

We had a preliminary discussion on it with Dr. Petri who is the chief of the division of Infectious Diseases and International Health at University of Virginia, who has expertise in this area of work, is a long-time icddr,b collaborator and is interested in this opportunity. We considered including the SNP analysis at a later date by amendment to the protocol, but decided to include our plan in the original protocol, as it is important we secure consent from the study participants for collecting their blood samples and storing the sample for further analysis. Thus we have made the necessary plans for sample collection and storage. We propose to store the blood sample so that we can use it in future and we are working on finalizing the SNP analysis plan and securing the necessary additional resources. We do believe though that it is important that we collect the necessary samples for this analysis, as these are samples we could not easily collect retrospectively from our study participants. We have not yet included Dr Petri's name in our investigators list, but will do so (via protocol amendment) once we have a concrete data analysis plan, an MOU between both institutions and the necessary resources.

## Sample Size Calculation and Outcome (Primary and Secondary) Variable(s)

| Clearly mention your assumptions. List the power and precision desired. Describe the optimal conditions to attain the sample size. Justify the sample size that is deemed sufficient to achieve the specific aims. |
| --- |

From the patients records who were admitted to the Matlab Hospital of icddr,b and calculated duration of severe pneumonia (mean=6 , standard deviation=2.5)^10^. To calculate sample size corresponding to the hypothesis that the Vitamin D supplement will reduce duration of severe pneumonia we assume the following things:

Mean duration in control group m1=6 days

Standard deviation in control group s1=2.5

Mean in Vitamin D group m2= a sequence of duration up to 5 for example, mean duration in this group is 1 or 2 or 3 or 4 or 5 days.

Standard deviation in Vitamin D group s2 = s1=2.5

Probability of Type-I error alpha= 0.05

Power = 90%

The Mathematical formula is for each group


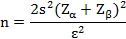


Where
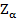
 and
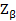
are standard normal deviate at 5% (alpha) and 10% (beta),
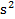
 is the common variance in this case this is equal to
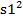
 and
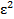
 is the squared difference between duration in both groups. Putting all the values we have


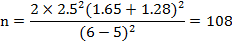


With above assumption we calculated as series of sample size and then consider 30% dropout in each case we adjust the calculated sample size. The following table gives the result of the sample size calculation.

| Mean in Duration in control group | Mean duration in Vitamin D group | Effect Size  in percentage | Standard deviation is both group | Initial sample size  in each group | Sample size after adjusting 30% dropout |
| --- | --- | --- | --- | --- | --- |
| 6 | 5 | 16.7 | 2.5 | 108 | 155 |
| 6 | 4 | 33.3 | 2.5 | 28 | 40 |
| 6 | 3 | 50.0 | 2.5 | 13 | 19 |
| 6 | 2 | 66.7 | 2.5 | 8 | 12 |
| 6 | 1 | 83.3 | 2.5 | 6 | 9 |

**Outcome measures/variables:**

Primary outcome measure will be time to resolution of severe pneumonia.

Secondary outcome measures will be duration of hospitalization, fever, tachypnoea, chest in drawing, hypoxia, lethargy and inability to feed during hospital stay and as well as new episode of pneumonia after discharge.

## Data Analysis

Data will be entered onto a personal computer (PC) and analysed by the principal investigator using STATA. We will perform the Chi-square test to compare categorical variables, and unpaired t test or ANOVA (Analysis of Variance) to compare quantitative variables. Intention to Treat (ITT) analyses will be done for assessing the impact of vitamin D supplementation on the outcome variable. To compare the median duration of each outcome variables Kaplan-Meier survival function plots will be done. To adjust the treatment effects for potential confounding factors and to evaluate the effect modification Cox proportional hazard regression model will be done. P value will be considered as significant when less than 0.05. Risk ratio (RR) will be estimated to compare the recovery rate in the two groups. Incidence rates of pneumonia will be calculated by dividing the number of new episodes of pneumonia by total time at risk for all children. Hazard ratios with 95% CIs will be obtained with Cox proportional-hazards models to measure time to repeat episodes between treatment groups.

## Data Safety Monitoring Plan (DSMP)

Data will be accessible to the PI, co-investigators, study physician and members of IRB. All the activities in this study will be closely monitored by PI and Co PIs. However a data safety and monitoring board (DSMB) will be there to meet at regular intervals for the monitoring of safety issue on vitamin D toxicity and any serious adverse event will be reported to the board.

## Ethical Assurance for Protection of Human rights

The study protocol and data collection tools, and the informed consent forms to be used in the study will be submitted to the Ethical Review Committee (ERC) of icddr,b, and the study would be initiated only after receiving approval of the committee. All medical information, description of treatment, and results of the laboratory tests performed will be kept under lock and key, only research staff, hospital physicians and nurses, and ERC/DSMB members of icddr,b will have access to these information.

## In this study we will see the impact of vitamin D on severe pneumonia among under five children. Although researchers first identified the fat-soluble vitamin D (cholecalciferol) almost a century ago, we know a little about mechanisms of vitamin D toxicity. Even with current data support the viewpoint that the biomarker plasma 25(OH)D concentration must rise above 750 nmol/L to produce vitamin D toxicity, the more prudent upper limit of 250 nmol/L might be retained to ensure a wide safety margin. Therefore for this study to ensure the safe dose of vitamin D3 for the children we will first proceed through piloting. We will observe any adverse event after the intervention and monitor the serum 25(OH)D level on admission, at discharge (day 5) and after two weeks of discharge as the half life of vitamin D3 is two weeks. We will also do serum calcium level on day of admission, 2^nd^ day and at discharge for safety monitoring as vitamin Ds primary roll is to control the levels of calcium in the body by constantly adding calcium to or taking calcium from bones. Depending on the findings of this pilot phase, we will decide the final dose for vitamin D3 intervention for the study. We will consider the toxic level of 25(OH)D as 250nmol/L^33,34^ and the upper limit of the serum calcium would be 2.63 mmol/L for safety monitoring.^35^ If we find serum 25(OH)D and calcium level on admission above this level we will stop further intervention.

For the main phase of the study we will do serum test for ionized calcium instead of total calcium.

As we have considered the upper normal limit of total serum calcium for safety monitoring, in case of ionized calcium the upper limit (normal range: 1.15-1.33 mmol/L) will be considered for safety monitoring. So the child having ionized calcium above 1.33mmol/L on admission will be stopped from having further intervention and excluded from the study.

## Use of Animals

The study will not involve the use of any animal.

## Collaborative Arrangements

Not applicable.

## Facilities Available

The study will be conducted at the Dhaka Hospital of the International Centre for Diarrhoeal Disease Research, Bangladesh (icddr,b), Dhaka, Bangladesh. This hospital has separate ward for management of children with severe pneumonia, well equipped laboratory facilities capable of performing all the clinical tests and serological measurements proposed in this study and also has an Intensive Care Unit, equipped with mechanical ventilation and other facilities of management of critically ill patients, as well as properly trained clinical staff to provide care to young patients suffering with severe pneumonia.

**Literature Cited**

1. Graham SM, English M, Hazir T, Enarson P & Duke T. Challenges to improving case management of childhood pneumonia at health facilities in resource-limited settings. Bulletin of the World Health Organisation 2008; 86: 349–355.
2. Rudan I, Boschi-Pinto C, Biloglare Z, Mulholland K, Campbell H. Epidemiology and etiology of childhood pneumonia. Bulletin World Health Organisation 2008; 86:408–416.
3. [Christa L Fischer Walker](http://www.thelancet.com/search/results?fieldName=Authors&searchTerm=Christa L Fischer+Walker), [Igor Rudan](http://www.thelancet.com/search/results?fieldName=Authors&searchTerm=Igor+Rudan), [Li Liu](http://www.thelancet.com/search/results?fieldName=Authors&searchTerm=Li+Liu), [Harish Nair](http://www.thelancet.com/search/results?fieldName=Authors&searchTerm=Harish+Nair), [Evropi Theodoratou](http://www.thelancet.com/search/results?fieldName=Authors&searchTerm=Evropi+Theodoratou), [Zulfiqar A Bhutta](http://www.thelancet.com/search/results?fieldName=Authors&searchTerm=Zulfiqar A+Bhutta), [Katherine L O'Brien](http://www.thelancet.com/search/results?fieldName=Authors&searchTerm=Katherine L+O'Brien), [Harry Campbell](http://www.thelancet.com/search/results?fieldName=Authors&searchTerm=Harry+Campbell), [Robert E Black](http://www.thelancet.com/search/results?fieldName=Authors&searchTerm=Robert E+Black). Global burden of childhood pneumonia and diarrhoea. The Lancet 2013; 381:9875, 1405-1416.
4. Arifeen SE, Akhter T, Chowdhury HR, Rahman KM, Chowdhury EK, Alam N   et al. Causes of death in children under five years of age. In: National Institute of Population Research and Training (NIPORT), Bangladesh Demographic and Health Survey 2004. Dhaka: National Institute of Population Research and Training, 2004; 125-33.
5. Chisti MJ, Tebruegge M, La Vincente S, Graham SM, Duke T. [Pneumonia in severely malnourished children in developing countries - mortality risk, aetiology and validity of WHO clinical signs: a systematic review](http://www.ncbi.nlm.nih.gov/pubmed/19772545). Trop Med Int Health 2009 Oct; 14(10):1173-89.
6. United State Agency for International Development. Acute Respiratory Infection (ARI) Programs. Global Health - Child Survival. Washington, DC: USAID; 2002.
7. UNICEF/WHO. Pneumonia: The forgotten killer of children. Geneva: WHO; 2006.
8. Vitamin A and Pneumonia Working Group. Potential interventions for the prevention of childhood pneumonia in developing countries: a meta-analysis of data from field trials to assess the impact of vitamin A supplementation on pneumonia morbidity and mortality. Bull. WHO 1995; 73: 609–619.
9. Zinc Investigators’ Collaborative Group. Prevention of diarrhea and pneumonia by zinc supplementation in children in developing countries: pooled analysis of randomized controlled trials. J. Pediatr 1999; 135: 689–697.
10. Brooks WA, Yunus M, Santoshan M, Wahed MA, Nahar K, Yeasmin S, *et al*. Zinc for severe pneumonia in very young children; double- blinded placebo- controlled trial. Lancet 2004; 363:1683-8.
11. Anuradha Bose, Christian L Coles, Gunavathi, Hemanth John, Prabhakar Moses, P Raghupathy et al. Efficacy of zinc in the treatment of severe pneumonia in hospitalized children <2 y old. American Journal of Clinical Nutrition 2006 May; 83 (5):1089-96.
12. Wayse V, Yousafzai A, Mogale K, Filteau S. Association of subclinical vitamin D deficiency with severe acute lower respiratory infection in Indian children under 5 years. Eur J Clin Nutr 2004; 58:563-7.
13. Monto AS, Lehmann D. Acute respiratory infections (ARI) in children: prospects for prevention. Vaccine 1998; 16:1582-8.
14. White HJ. Vitamin D signaling, infectious diseases and regulation of innate immunity. Infection and Immunity 2008; 76: 3837–3843.
15. Pichler J, Gerstmyr M, Szepfalusi Z, Urbanek R, Peterlik M & Willheim M. 1-alpha, 25(OH)2D3 inhibits not only Th1 but also Th2 differentiation in human cord blood T cells. Pediatric Research 2002; 52: 12–18.
16. Cantorna MT. Vitamin D and autoimmunity: is vitamin D status an environmental factor affecting autoimmune disease prevalence? Proceedings of the Society for Experimental Biology and Medicine 2000; 223: 230–233.
17. Rockett KA, Brookes R, Udalova I, Vidal V, Hill AV & Kwiatkowski D. 1,25-Dihydroxyvitamin D3 induces nitric oxide synthase and suppresses growth of Mycobacterium tuberculosis in a human macrophage-like cell line. Infection and Immunity 1998; 66: 5314–5321.
18. Von Essen MR, Kongsbak M, Schjerling P, Olgaard K, Niels O, Geisler C. **Vitamin D controls T cell antigen receptor signaling and activation of human T cells.** Nature Immunology, 2010; 11:344-9
19. Muhe L, Lulseged S, Mason KE, Simoes EAF. Case control study of the role of nutritional rickets in the risk of developing pneumonia in Ethiopian children. Lancet 1997; 349:1801-4.
20. Banajeh SM. Nutritional rickets and vitamin D deficiency—association with the outcomes of childhood very severe pneumonia: A prospective cohort study. Pediatric Pulmonology 2009; 44:1207–1215.
21. Salimpour R. Rickets in Tehran. Arch Dis Child 1975; 50:63-5.
22. Lubani MM, Al-Sheb TS, Sharda DC, Quattawi SA, Ahmed SAH, Moussa MA, *et al*. Vitamin-D deficiency rickets in Kuwait: the prevalence of preventable disease. Ann Trop Paediatr 1989; 3:134-9.
23. Lulseged S. Severe rickets in a children hospital in Addis Ababa. Ethiopia Med J 1990; 28:175-81.
24. [Abdelhamid S. Najada](http://tropej.oxfordjournals.org/search?author1=Abdelhamid+S.+Najada&sortspec=date&submit=Submit), [Moen S. Habashneh](http://tropej.oxfordjournals.org/search?author1=Moen+S.+Habashneh&sortspec=date&submit=Submit), [Maher Khader](http://tropej.oxfordjournals.org/search?author1=Maher+Khader&sortspec=date&submit=Submit). The Frequency of Nutritional Rickets among Hospitalized Infants and its Relation to Respiratory Diseases. Journal of Trop Pediatr 2004; 50 (6): 364-368.
25. Peter Bergman, Asa U. Lindh, Linda Bjorkhem-Bergman, Jonatan D. Lindh. Vitamin D and Respiratory Tract Infections: A Systematic Review and Meta-Analysis of Randomized Controlled Trials. PLOS ONE June 2013; 8 (6). doi:10.1371/journal.pone.0065835
26. Semira Manaseki-Holland, Ghulam Qader, Mohammad Isaq Masher, Jane Bruce, M. Zulf Mughal, Daniel Chandramohan and Gijs Walraven. Effects of vitamin D supplementation to children diagnosed with pneumonia in Kabul: a randomised controlled trial. Tropical Medicine and International Health , 2010 Oct; 15 (10):1148–1155.
27. Nidhi Choudhary Piyush Gupta. Vitamin D Supplementation for Severe Pneumonia – A Randomized Controlled Trial. Indian Pediatrics June 2012; vol:49
28. Fischer PR, Rahman A, Cimma JP, Kyaw-Myint TO, Kabir AR, Talukder, *et al*. Nutritional rickets without vitamin Ddeficiency in Bangladesh. J Trop Pediatr 1999; 45: 291-3.
29. Daniel E. Roth, M. Rashed Shah, Robert E. Black, and Abdullah H. Baqui. Vitamin D Status of Infants in Northeastern Rural Bangladesh: Preliminary Observations and a Review of Potential Determinants. Journal of Health Population and Nutrition 2010 Oct;28(5):458-469.

# [**Chisti MJ**](http://www.ncbi.nlm.nih.gov/pubmed?term=Chisti MJ%5BAuthor%5D&cauthor=true&cauthor_uid=22041465), [**Duke T**](http://www.ncbi.nlm.nih.gov/pubmed?term=Duke T%5BAuthor%5D&cauthor=true&cauthor_uid=22041465), [**Robertson CF**](http://www.ncbi.nlm.nih.gov/pubmed?term=Robertson CF%5BAuthor%5D&cauthor=true&cauthor_uid=22041465), [**Ahmed T**](http://www.ncbi.nlm.nih.gov/pubmed?term=Ahmed T%5BAuthor%5D&cauthor=true&cauthor_uid=22041465), [**Faruque AS**](http://www.ncbi.nlm.nih.gov/pubmed?term=Faruque AS%5BAuthor%5D&cauthor=true&cauthor_uid=22041465), [**Bardhan PK**](http://www.ncbi.nlm.nih.gov/pubmed?term=Bardhan PK%5BAuthor%5D&cauthor=true&cauthor_uid=22041465), [**La Vincente S**](http://www.ncbi.nlm.nih.gov/pubmed?term=La Vincente S%5BAuthor%5D&cauthor=true&cauthor_uid=22041465), [**Salam MA**](http://www.ncbi.nlm.nih.gov/pubmed?term=Salam MA%5BAuthor%5D&cauthor=true&cauthor_uid=22041465). Co-morbidity: exploring the clinical overlap between pneumonia and diarrhoea in a hospital in Dhaka, Bangladesh. [**Ann Trop Paediatr.**](http://www.ncbi.nlm.nih.gov/pubmed/22041465) 2011;31(4):311-9.

# [**Walker CL**](http://www.ncbi.nlm.nih.gov/pubmed?term=Walker CL%5BAuthor%5D&cauthor=true&cauthor_uid=23826506)^1^, [**Perin J**](http://www.ncbi.nlm.nih.gov/pubmed?term=Perin J%5BAuthor%5D&cauthor=true&cauthor_uid=23826506), [**Katz J**](http://www.ncbi.nlm.nih.gov/pubmed?term=Katz J%5BAuthor%5D&cauthor=true&cauthor_uid=23826506), [**Tielsch JM**](http://www.ncbi.nlm.nih.gov/pubmed?term=Tielsch JM%5BAuthor%5D&cauthor=true&cauthor_uid=23826506), [**Black RE**](http://www.ncbi.nlm.nih.gov/pubmed?term=Black RE%5BAuthor%5D&cauthor=true&cauthor_uid=23826506). **Diarrhea** as a risk factor for acute lower respiratory tract infections among young children in low income settings. [**Walker CL**](http://www.ncbi.nlm.nih.gov/pubmed?term=Walker CL%5BAuthor%5D&cauthor=true&cauthor_uid=23826506), [**Perin J**](http://www.ncbi.nlm.nih.gov/pubmed?term=Perin J%5BAuthor%5D&cauthor=true&cauthor_uid=23826506), [**Katz J**](http://www.ncbi.nlm.nih.gov/pubmed?term=Katz J%5BAuthor%5D&cauthor=true&cauthor_uid=23826506), [**Tielsch JM**](http://www.ncbi.nlm.nih.gov/pubmed?term=Tielsch JM%5BAuthor%5D&cauthor=true&cauthor_uid=23826506), [**Black RE**](http://www.ncbi.nlm.nih.gov/pubmed?term=Black RE%5BAuthor%5D&cauthor=true&cauthor_uid=23826506).

1. Mohammad Tahazzul, Shakal N. Singh. Does Vitamin D supplementation benefit children with severe pneumonia? Critical appraisal of article Vitamin D supplementation for severe pneumonia - a randomized controlled trial. clinical epidemiology and global health 1 (2013) 37 -39.
2. Glenville Jones. Pharmacokinetics of vitamin D toxicity. American J Clinical Nutrition, 2008;88:582S–6S.
3. Madhusmita Misra, Danièle Pacaud, Anna Petryk, Paulo Ferrez Collett-Solberg, Michael Kappy and on behalf of the Drug and Therapeutics Committee of theWilkins Pediatric Endocrine Society. Vitamin D Deficiency in Children and Its Management: Review of Current Knowledge and Recommendations. Pediatrics 2008;122;398-417.
4. Daniel E Roth, Abdullah Al Mahmud, Rubhana Raqib, Evana Akhtar, Nandita Perumal, Brendon Pezzack, Abdullah H Baqui. Randomized placebo-controlled trial of high-dose prenatal third-trimester vitamin D3 supplementation in Bangladesh: the AViDD trial. Nutrition Journal 2013, 12:47.
5. Pocket book of Hospital care for children: Guidelines for the management of common childhood illnesses. Second edition, 2013. WHO, Geneva.
6. Hasan Ashraf, Mohammod Jobayer Chisti and Nur Haque Alam. Treatment of childhood pneumonia in developing countries. Health Management. Krzysztof Smigórski, 2010 (59-88).
7. Tietz NW. Fundamentals of Clinical Chemistry; 6^th^ edition, Philadelphia: WB Saunders Co.; 2008: 840p.
8. http://en.wikipedia.org/wiki/Hypervitaminosis_D

**ANNEXURE**

**ANNEXURE-1-A: Voluntary Consent Form for Piloting (English)**

| Protocol No. 13088 | Version No. 1.00 | Date: 24-10-2013 |
| --- | --- | --- |

Protocol Title: Vitamin D supplementation: Impact on severe pneumonia among under-five children

Investigator’s name: Fahmida Chowdhury

Organization: icddr,b

**Purpose of the research**

**Background**

Your child is suffering from severe pneumonia, which is the leading cause of morbidity among under-five children, in developing countries including Bangladesh. Such children require admission to a hospital for treatment and care for at least five days.

There is a micronutrient known as Vitamin D. We think addition of vitamin D in the routine management of children, as stated above, may help faster recovery of children from severe pneumonia and shorten the duration of their hospital stay. However, we need to conduct a research study to determine if treatment with vitamin D could actually help earlier recovery of children with severe pneumonia and thus their earlier discharge from the hospital. We are conducting such a research study at this hospital on 350 under-five children with severe pneumonia. Before conducting the study we are intending to determine the safe dose of vitamin D intervention for the children through a pilot study. There are some studies in other countries where they have used high dose of vitamin D in children without showing any side effect. For piloting, comparing to those studies we are using two separate lower dose schedule in two groups of children to understand which dose schedule will be better for the children for the main study. For this purpose we are enrolling a total of twenty children in two groups i.e. ten children in each group. Children in each group will receive vitamin D in two different doses.

**Why invited to participate in the study?**

We need to enrol children under five years of age with severe pneumonia to test which dose of vitamin D would be safe in their treatment. As your child is suffering from severe pneumonia, we request you to help us by allowing enrolment of your child in this study.

**Methods and procedures**

If you allow participation of your child in our study, you may expect the followings:

- We would admit your child to the Respiratory Ward of this hospital, arrange for her/his appropriate treatment in accordance with guidelines of this hospital, and keep your child admitted until she/he recovers from pneumonia.
- We would ask you some questions related to your child’s illness, and perform her/his thorough physical examinations at the time of admission and on each day of hospitalisation to assess progress of illness (improvement or deterioration).
- All children, including your child, will receive the standard good treatment and care of this hospital.
- In addition to chest X-ray we will collect 5.0 mL (about one teaspoonful) of blood from a suitable

vein on your child’s arm for blood test on day of admission, 1ml of blood on 2^nd^ day, and 2 ml of blood on day of discharge and after two weeks of discharge. We have to assess vitamin D and calcium level in blood of your child through these tests to measure the safety level. We are requesting you to bring your child for follow-up after two weeks of discharge from hospital and we will collect 2.0 ml blood from your child at that time for test.

- Ten children from each group will receive vitamin D in two different doses in addition to the standard management of this hospital. Vitamin D will be administered by mixing that in expressed breast milk of the mothers/infant formula for infants younger than six months, and by mixing that in diets (such as milk suji) if the child is 6 months of age or older.

**Risk and benefits**

With the exception of receiving vitamin D, the treatment and care of the children will not differ from what would be routinely done at this hospital. We will use doses of vitamin D that is expected to be safe. We will regularly monitor your child’s clinical condition with testing serum vitamin D and calcium level. If serum vitamin D or calcium level is found to be higher than the normal range or any symptom of hypervitaminosis develops we will immediately stop further intervention so that your child may not have any adverse effect and treat accordingly. Collection of 5.0 mL of blood for this research study will not cause any harm to your child, except that she/he will feel momentary pain from the needle stick. There is rare chance of temporary discolouration of skin surrounding the needle prick, and very rare chance of infection at the prick site and/or in the blood. However, we will take necessary precautions to prevent such problems, including undertaking of sterile precautions using disposable syringes and needles. We will provide best possible treatment at this hospital, at no cost to you, in the event of any infection.

However, with the exception of more close observation by the research team and the standard treatment of this hospital, your child may not receive any other benefit from participation in this study. We are also not certain about the benefits of vitamin D in the treatment of severe pneumonia. The results of this study would improve our knowledge, and if vitamin D is proved to be beneficial that would improve management of children with severe pneumonia in the future, benefitting millions of children in Bangladesh and globally every year.

**Privacy, anonymity and confidentiality**

We do hereby affirm that privacy, anonymity and confidentiality of data/information identifying your child will strictly be maintained. We would keep all medical information, description of treatment, and results of the laboratory tests performed on your child confidential, under lock and key, and none other than our research staff will have an access to these information.

**Future use of information**

In future, information collected from you may be used by another researcher. But in that case privacy, anonymity and confidentiality of information would be maintained in such way that it would not identify you.

**Right not to participate and withdraw**

Participation of your child in this study is voluntary. Refusal to take part in the study will involve no penalty and your child will receive the standard treatment of this hospital. You have right to withdraw your child at any time from the study and you also have right to refuse answering any question.

**Principle of compensation**

Treatment at this hospital is free for all patients, and your child will not be any exception. Similarly, we will not pay money for participation of your child in our study. You will only receive the transport cost for follow up visit of your child.

We will happily provide you further information about the study, if any, now or at a later time. You may communicate with the principal investigators of the study at the contact address given below. We will answer to your question related to your patent’s medical condition, treatment, and results of any or all tests performed on your patient. However, we would like to inform you that some of the tests will be performed at the end of the study, and thus their results would be available only then.

If you agree to our proposal of enrolling your child in our study, please indicate that by putting your signature or your left thumb impression at the specified space below

Thank you for your cooperation

_______________________________________ ____________________

Signature or left thumb impression of Date

Parent/ Guardian/ Attendant

_______________________________________ ____________________

Signature or left thumb impression of the witness Date

_______________________________________ ___________________

Signature of the PI or his/her representative Date

# (NOTE: In case of representative of the PI, she/he shall put her/his full name and designation and then sign)

# (Name and contact phone of IRB Secretariat, RA, M. A. Salam Khan, Phone No: 9886498 or PABX 8860523-32 Extension. 3206; Principal Investigator, Dr. Fahmida Chowdhury, Phone No. PABX 8860523-32 Extension. 2550).

**ANNEXURE-2: CASE REPORT FORM (CRF)**

**Vitamin D supplementation: Impact on severe pneumonia among under-five children**

Child’s name: ………………………………………………………………………………………………

Father’s name: ……………………………………………………………………………………………..

Address: ……………………………………………………………………………………………………..

…………………………………………………………………………………………………….

Phone number: ………………………………………………………………………………………………

**Socio-demographic History:**  **Code**

1. Study ID number
2. Date of admission

3. Date of enrolment

4. Date of discharge

5. Hospital Registration No:

6. Allocation number

7. Allocation group:1=A, 2=B

# 8. Place of normal residence of the patient

(1=within Dhaka city, 2=within Dhaka district, 3= outside Dhaka district)

9. Age (in months)

10. Sex: 1=Male , 2= Female

11. Religion of the patient

(1= Muslim, 2=Hindu, 3=Christian, 4=Buddhist, 5= Others)

12. Gestational age at birth (weeks) [ code 99, if not known)

13. Father’s age (in years)

14. Mother’s age (in years)

15. Education of Patient’s Father

(1=none, 2=maktab, 3=1-5 yrs of schooling, 4=6-10 yrs, 5=10-12 yrs, 6= >12 yrs of schooling)

16. Education of Patient’s Mother

(0=none, 1=maktab, 2=1-5 yrs of schooling

3=6-10 yrs, 4=10-12 yrs, 5= >12 yrs of schooling)

17. Father’s occupation

(1=unemployed, 2= day labourer, 3=hawker, 4=petty business

5= garments worker, 6=private job, 7= govt.job, 8=driver, 9=rickshawpuller)

18. Mother’s occupation

(1=house wife, 2= garments worker, 3= day labourer,

4= private job, 5= govt. job, 6= maid servant, 7=street begger)

19. Family History of Tuberculosis

(1=yes, 2=no)

20. Family History of respiratory tract infection (within past 14days) (1=yes, 2=no)

21. Past History of pneumonia

(1=no pneumonia, 2=1 time, 3=2 times, 4=>2 times)

22. Breast feeding

(1=yes, 2=no, 9=not applicable)

23. Duration of breast feeding (in months)

24. Exclusive breast feeding

(1=yes, 2=no, 9=not applicable)

25. If non breast fed then type of feed

(1=cereal, 2=formula milk, 3=cow’s milk, 4=others)

26. Duration of complementary feeding (in months)

27. Number of rooms in the house hold

28. Number of adults sharing the same sleeping area with the child

29. Number of children sharing the same sleeping area with the child

30. Cooks inside bedroom

(1=yes, 2=no)

31. Monthly income (BDT)

32. History of smoking in the family

(1=none smokes, 2=father smokes, 3=mother smokes, 4=both parents smoke

5= other family member smokes)

33. Type of locality where care giver live?

(1=slum, 2=common housing area, 3= residential area, 4= village, 5= others)

34. Dwelling status (sun exposure)

(1=not at all, 2=mild, 3=moderate, 4=adequate)

35. How much time spends at outdoor daily (in hours)?

36. Percentage of body covered with clothes when outdoor (%)

[according to “rule of nines”- head and neck (18%), body (36%),

upper limb (18%), lower limb (28%), genitalia (1%)]

37. Number of siblings

38. Birth order of the diseased child

39. Number of under-five children in the family other than the diseased child

40. How many people live in the family?

41.Immunization status

BCG 1= Yes, 2=No , 3=Don’t know, 9=NA

Penta 1 1= Yes, 2=No, 3=Don’t know, 9=NA

Penta 2 1= Yes, 2=No, 3=Don’t know , 9=NA

Penta 3 1= Yes, 2=No, 3=Don’t know, 9=NA

Oral polio 4 1=Yes, 2=No, 3=Don’t know, 9=NA

MR 1= Yes, 2=No, 3=Don’t know, 9=NA

Measles 2 1=Yes, 2=No, 3=Don’t know, 9=NA

42. Whom did you consulted first for this episode of illness of your child?

(1= qualified doctor, 2= homeopath doctor 3= spiritual adviser

4= quack, 5= drug seller, 6=traditional healer, 7= others, 8=none)

43. Why delay (if any)?

(1= financial constraint, 2= to take care at home, 3= under treatment of quack

4= under homoeopath or spiritual treatment, 5= could not realise the problem

6= single at home, 7= others, 9=not applicable)

44. a. Use of antibiotics before admission in hospital

(1=yes, 2=no, 3=don’t know, 4=not applicable)

b. Name of antibiotic used before admission in hospital

(1=Amoxycillin, 2=Cefixime, 3=Azythromycin, 4=Ciprofloxacin, 5=Ceftriaxone

6=Cefpodoxime, 9=Don’t know)

45**. Symptoms on admission**

| **Symptoms** | **1 = yes / 2 = no** | **Duration in days** |
| --- | --- | --- |
| Diarrhoea  (Type of diarrhea:1=AWD,2=ID,3=PD) |  |  |
| Cough |  |  |
| Running nose |  |  |
| Fever |  |  |
| Vomiting |  |  |
| Respiratory distress |  |  |
| Poor oral intake |  |  |
| Lethargy |  |  |
| Convulsion |  |  |
| Others 1 |  |  |
| Others 2 |  |  |
| Others 3 |  |  |
| Others 4 |  |  |

46.**Clinical Examination**

**A) ANTHROPOMETRY**

- Admission height/length (cm)
- Admission weight (kg)
- W/A%
- ZW (z score)
- W/L%
- WL (z score)

- L/A%
- LA (z score)

**B) General examination**

- Radial pulse (rate/min) [ If imperceptible, code “000”]
- Respiratory rate (per minute)
- Axillary temperature (°C)
- Pallor

(1=yes, 2=no)

- Cyanosis

(1=yes, 2=no)

- Icterus

(1=yes, 2=no)

- Clubbing

(1=yes, 2=no)

- Pedal edema

(1=yes, 2=no)

- Lymphadenopathy

(1=yes, 2=no)

- Spo2 (%)
- Dehydration

(1=no sign, 2=some, 3=severe, 9=not applicable)

**C) Systemic examination**

1. **Respiratory system**

- Lower chest wall in drawing 1=yes, 2=no
- Nasal flaring 1=yes, 2=no

- Central cyanosis 1=yes, 2=no
- Grunting 1=yes, 2=no
- Head nodding 1=yes, 2=no
- Crackles 1=yes, 2=no
- Rhonchi 1=yes, 2=no
- Bronchial breathing 1=yes, 2=no
- Wheezing 1=yes, 2=no

1. **Cardiovascular system**

- Murmur

(1=yes, 2=no)

c**) Abdominal system**

- Liver palpable

(1=yes, 2=no)

- Spleen palpable

(1=yes, 2=no)

**d) Central nervous system**

Glasgow coma scale(GCS)

Physician’s initials: ____________ Date: ___/___/_____

47**. Laboratory investigations**

- Total WBC/cu.mm (Not done= 99)
- Poly% (Not done= 99)
- Lymphocytes (%) (Not done= 99)
- Immature poly (Band) % (Not done= 99)
- Hct% (Not done= 99)
- Sodium; mmol/L (Not done= 99)
- Potassium; mmol/L (Not done= 99)
- Chloride; mmol/L (Not done= 99)
- TCO_2_; mmol/L (Not done= 99)
- HCO3; mmol/L (Not done= 99)

- C-reactive protein on admission (Not done= 99)
- Blood glucose on admission(mmol/L) (Not done= 99)
- Serum “25 (OH)-D” level on admission(nmol/L) (Not done= 99)
- Serum “25 (OH)-D” level on discharge(nmol/L) (Not done= 99)
- Serum ionized Ca on admission(mmol/L) (Not done= 99)
- Serum ionized Ca on discharge(mmol/L) (Not done= 99)
- Serum phosphorus on admission(mmol/L) (Not done= 99)
- Serum Alkaline phosphatase on admission(U/L) (Not done= 99)
- Serum PTH on admission (pmol/L) (Not done= 99)
- Serum PTH at discharge (pmol/L) (Not done= 99)
- Blood culture 1=Positive, 2= Negative, 3=Not done

- Blood culture isolate :

(1=Streptococcus pneumoniae, 2=Staphylococcus aureus, 3=Haemophylus influenzae, 4=Salmonella typhi, 5=Salmonella para-typhi, 6=Non typhoidal Salmonella, 7=E. Coli, 8=Klebsiella, 9=Enterococcus, 10=Acinetobacter, 11=Pseudomonas,12=CNS, 13=Enterobacter species , 14=Poly microbial)

- Chest X-ray findings:

(1=normal, 2=primary end point consolidation, 3=other consolidation/infiltrate,

4=pleural effusion, 5=pneumothorax)

48**. Diet with Vitamin D3:/ Diet without Vitamin D3:**

| Day | Date | Time | Dose given | If any vomiting within 30 mins, repeat the dose | If any adverse effect* |
| --- | --- | --- | --- | --- | --- |
| 1. |  |  |  |  |  |
| 2. |  |  |  |  |  |
| 3. |  |  |  |  |  |
| 4. |  |  |  |  |  |
| 5. |  |  |  |  |  |

(*Adverse effect: vomiting, decreased appetite, irritability, constipation, dehydration, fatigue, muscle weakness)

49. **Antibiotics given:**

| Antibiotics | 1=yes,2=no | Day started | Day stopped | Comments |
| --- | --- | --- | --- | --- |
| Ampicilin |  |  |  |  |
| Gentamicin |  |  |  |  |
| Ceftriaxone |  |  |  |  |
| Levofloxacin |  |  |  |  |
| Ceftazidime |  |  |  |  |
| Amikacin |  |  |  |  |
| Flucloxacillin |  |  |  |  |
| Vancomycin |  |  |  |  |
| Imipenem |  |  |  |  |
| Meropenem |  |  |  |  |
| Cotrimoxazole |  |  |  |  |
| Ciprofloxacin |  |  |  |  |
| Clarithromycin |  |  |  |  |

50. The amount of vitamin D received through dietary source (IU)

(Infant formula/milk siji)

51. Final Outcome

- - - 1. Well & discharged
      2. Did not improve but discharged on request
      3. Died in hospital
      4. DORB
      5. Absconded
      6. Referred to another hospital

52. Duration of hospitalization (in days)

Physician’s initials: ___________________ Date: ___/___/____

**Follow up During Hospital Stay**

| Days | Date | Time | Pulse/min | Resp rate  /min | Chest indrawing | Spo2  (%) | | Temp  (◦C) | Lungs  (1=fine creps  2=coarse creps  3=clear) | Cough | Feeding  (1=well 2=poor) | Lethargy | Convulsion | AWD | Sign symtomps of Raised ICP | Pneumonia status^*^ |
| --- | --- | --- | --- | --- | --- | --- | --- | --- | --- | --- | --- | --- | --- | --- | --- | --- |
| **Day 1** |  |  |  |  |  |  |  | |  |  |  |  |  |  |  |  |
| 0 hrs |  |  |  |  |  |  |  | |  |  |  |  |  |  |  |  |
| 8 hrs |  |  |  |  |  |  |  | |  |  |  |  |  |  |  |  |
| 16 hrs |  |  |  |  |  |  |  | |  |  |  |  |  |  |  |  |
| 24 hrs |  |  |  |  |  |  |  | |  |  |  |  |  |  |  |  |
| **Day 2** |  |  |  |  |  |  |  | |  |  |  |  |  |  |  |  |
| 8hrs |  |  |  |  |  |  |  | |  |  |  |  |  |  |  |  |
| 16 hrs |  |  |  |  |  |  |  | |  |  |  |  |  |  |  |  |
| 24 hrs |  |  |  |  |  |  |  | |  |  |  |  |  |  |  |  |
| **Day 3** |  |  |  |  |  |  |  | |  |  |  |  |  |  |  |  |
| 8 hrs |  |  |  |  |  |  |  | |  |  |  |  |  |  |  |  |
| 16 hrs |  |  |  |  |  |  |  | |  |  |  |  |  |  |  |  |
| 24 hrs |  |  |  |  |  |  |  | |  |  |  |  |  |  |  |  |
| **Day 4** |  |  |  |  |  |  |  | |  |  |  |  |  |  |  |  |
| 8hrs |  |  |  |  |  |  |  | |  |  |  |  |  |  |  |  |
| 16 hrs |  |  |  |  |  |  |  | |  |  |  |  |  |  |  |  |
| 24 hrs |  |  |  |  |  |  |  | |  |  |  |  |  |  |  |  |
| **Day 5** |  |  |  |  |  |  |  | |  |  |  |  |  |  |  |  |
| 8hrs |  |  |  |  |  |  |  | |  |  |  |  |  |  |  |  |
| 16hrs |  |  |  |  |  |  |  | |  |  |  |  |  |  |  |  |
| 24 hrs |  |  |  |  |  |  |  | |  |  |  |  |  |  |  |  |

(Pneumonia status^*^ 1=severe pneumonia continues, 2=severe pneumonia resolved, 3=pneumonia continues, 4=pneumonia resolved)

| Days | Date | Time | Pulse/min | Resp rate/min | Chest indrawing | Spo2  (%) | Temp  (◦C) | Lungs  (1=fine creps  2=coarse creps  3=clear) | Cough | Feeding  (1=well 2=poor) | Lethargy | Convulsion | AWD | Sign symtomps of Raised ICP | Pneumoia status* |
| --- | --- | --- | --- | --- | --- | --- | --- | --- | --- | --- | --- | --- | --- | --- | --- |
| **Day 6** |  |  |  |  |  |  |  |  |  |  |  |  |  |  |  |
| 0 hrs |  |  |  |  |  |  |  |  |  |  |  |  |  |  |  |
| 8 hrs |  |  |  |  |  |  |  |  |  |  |  |  |  |  |  |
| 16 hrs |  |  |  |  |  |  |  |  |  |  |  |  |  |  |  |
| 24 hrs |  |  |  |  |  |  |  |  |  |  |  |  |  |  |  |
| **Day 7** |  |  |  |  |  |  |  |  |  |  |  |  |  |  |  |
| 8hrs |  |  |  |  |  |  |  |  |  |  |  |  |  |  |  |
| 16 hrs |  |  |  |  |  |  |  |  |  |  |  |  |  |  |  |
| 24 hrs |  |  |  |  |  |  |  |  |  |  |  |  |  |  |  |
| **Day 8** |  |  |  |  |  |  |  |  |  |  |  |  |  |  |  |
| 8 hrs |  |  |  |  |  |  |  |  |  |  |  |  |  |  |  |
| 16 hrs |  |  |  |  |  |  |  |  |  |  |  |  |  |  |  |
| 24 hrs |  |  |  |  |  |  |  |  |  |  |  |  |  |  |  |
| **Day 9** |  |  |  |  |  |  |  |  |  |  |  |  |  |  |  |
| 8hrs |  |  |  |  |  |  |  |  |  |  |  |  |  |  |  |
| 16 hrs |  |  |  |  |  |  |  |  |  |  |  |  |  |  |  |
| 24 hrs |  |  |  |  |  |  |  |  |  |  |  |  |  |  |  |
| **Day10** |  |  |  |  |  |  |  |  |  |  |  |  |  |  |  |
| 8hrs |  |  |  |  |  |  |  |  |  |  |  |  |  |  |  |
| 16hrs |  |  |  |  |  |  |  |  |  |  |  |  |  |  |  |
| 24 hrs |  |  |  |  |  |  |  |  |  |  |  |  |  |  |  |

(Pneumonia status^*^ 1=severe pneumonia continues, 2=severe pneumonia resolved, 3=pneumonia continues, 4=pneumonia resolved)

**Follow up at home after discharge**

| **Weeks** | **Date** | **No ARI illness** | **No pneumonia (cough and cold)** | **Pneumonia** | **Severe pneumonia** | **Other illness** | **Final Outcome** |
| --- | --- | --- | --- | --- | --- | --- | --- |
| 1^st^ week |  |  |  |  |  |  |  |
| 2^nd^ week |  |  |  |  |  |  |  |
| 3^rd^ week |  |  |  |  |  |  |  |
| 4^th^ week |  |  |  |  |  |  |  |
| 5^th^ week |  |  |  |  |  |  |  |
| 6^th^ week |  |  |  |  |  |  |  |
| 7^th^ week |  |  |  |  |  |  |  |
| 8^th^ week |  |  |  |  |  |  |  |
| 9^th^ week |  |  |  |  |  |  |  |
| 10^th^ week |  |  |  |  |  |  |  |
| 11^th^ week |  |  |  |  |  |  |  |
| 12^th^ week |  |  |  |  |  |  |  |
| 13^th^ week |  |  |  |  |  |  |  |
| 14^th^ week |  |  |  |  |  |  |  |
| 15^th^ week |  |  |  |  |  |  |  |
| 16^th^ week |  |  |  |  |  |  |  |
| 17^th^ week |  |  |  |  |  |  |  |
| 18^th^ week |  |  |  |  |  |  |  |
| 19^th^ week |  |  |  |  |  |  |  |
| 20^th^ week |  |  |  |  |  |  |  |
| 21^st^ week |  |  |  |  |  |  |  |
| 22^nd^ week |  |  |  |  |  |  |  |
| 23^rd^ week |  |  |  |  |  |  |  |
| 24^th^ week |  |  |  |  |  |  |  |

**Standard Management Guidelines for Dhaka Hospital (Annexure 5)**
